# The Combined Expression of the Nonstructural Protein NS1 and the N-Terminal Half of NS2 (NS2_1-180_) by ChAdOx1 and MVA Confers Protection against Clinical Disease in Sheep upon Bluetongue Virus Challenge

**DOI:** 10.1128/JVI.01614-21

**Published:** 2022-02-09

**Authors:** Sergio Utrilla-Trigo, Luis Jiménez-Cabello, Eva Calvo-Pinilla, Alejandro Marín-López, Gema Lorenzo, Pedro Sánchez-Cordón, Sandra Moreno, Julio Benavides, Sarah Gilbert, Aitor Nogales, Javier Ortego

**Affiliations:** a Centro de Investigación en Sanidad Animal (CISA), Instituto Nacional de Investigación y Tecnología Agraria y Alimentaria (INIA-CSIC), Valdeolmos, Spain; b Section of Infectious Diseases, Department of Internal Medicine, Yale University School of Medicine, New Haven, Connecticut, USA; c Instituto de Ganadería de Montaña (CSIC-Universidad de León), León, Spain; d The Jenner Institute, University of Oxford, Oxford, UK; University of Kentucky College of Medicine

**Keywords:** bluetongue, NS1, NS2, MVA, multiserotype, DIVA, vaccine, CD8^+^ T cell response, bluetongue virus, orbiviruses, vaccines

## Abstract

Bluetongue, caused by bluetongue virus (BTV), is a widespread arthropod-borne disease of ruminants that entails a recurrent threat to the primary sector of developed and developing countries. In this work, we report modified vaccinia virus Ankara (MVA) and ChAdOx1-vectored vaccines designed to simultaneously express the immunogenic NS1 protein and/or NS2-Nt, the N-terminal half of protein NS2 (NS2_1-180_). A single dose of MVA or ChAdOx1 expressing NS1-NS2-Nt improved the protection conferred by NS1 alone in IFNAR(-/-) mice. Moreover, mice immunized with ChAdOx1/MVA-NS1, ChAdOx1/MVA-NS2-Nt, or ChAdOx1/MVA-NS1-NS2-Nt developed strong cytotoxic CD8^+^ T-cell responses against NS1, NS2-Nt, or both proteins and were fully protected against a lethal infection with BTV serotypes 1, 4, and 8. Furthermore, although a single immunization with ChAdOx1-NS1-NS2-Nt partially protected sheep against BTV-4, the administration of a booster dose of MVA-NS1-NS2-Nt promoted a faster viral clearance, reduction of the period and level of viremia and also protected from the pathology produced by BTV infection.

**IMPORTANCE** Current BTV vaccines are effective but they do not allow to distinguish between vaccinated and infected animals (DIVA strategy) and are serotype specific. In this work we have develop a DIVA multiserotype vaccination strategy based on adenoviral (ChAdOx1) and MVA vaccine vectors, the most widely used in current phase I and II clinical trials, and the conserved nonstructural BTV proteins NS1 and NS2. This immunization strategy solves the major drawbacks of the current marketed vaccines.

## INTRODUCTION

Bluetongue (BT) is an arthropod-borne disease of ruminants transmitted during blood-feeding by some species of biting midges of the genus *Culicoides* (1). This World Organization for Animal Health (OIE)-listed disease is characterized by a highly variable clinical spectrum. Sheep and some species of wild ruminants are the most affected hosts whereas cattle, goats, and the majority of wild ruminant species are usually asymptomatic (2). Bluetongue virus (BTV), the etiological agent of this severe livestock disease, belongs to the genus *Orbivirus*, which is included within the family *Reoviridae*. This nonenveloped virus presents a structure characterized by its icosahedral capsid (∼90 nm in diameter), which is divided in three concentric layers (3). The viral genome (∼19.2 kb) consists of 10 linear double-stranded RNA (dsRNA) segments (S1 to S10) that encode for seven structural proteins (VP1 to VP7) together with five nonstructural proteins (NS1, NS2, NS3/NS3A, NS4, and the putative protein NS5) (4, 5). VP2, involved in virus-entry and conforming the outer capsid layer together with VP5, is the main inductor of neutralizing antibodies (nAbs) and defines the serotype (6). To date, 29 serotypes of BTV have been identified, being classified as typical (1–24), and atypical (25–27), with two more putative serotypes, BTV-28 and BTV-29, recently described and very similar to other typical BTV serotypes (7–9). Despite BT has been historically prevalent in tropical and subtropical regions located between 35° S and 45° N, serotypes 1, 2, 4, 6, 8, 9, 11, and 16 have invaded Europe and other continents since 1998, leading to a global economic impact estimated in ∼$3 billion per year (10).

Due to the lack of therapeutic treatments (11, 12), vaccination against BTV constitutes the most effective prophylactic measure for BT control. Traditionally, vaccination strategies against this disease are based on conventional approaches, such as inactivated or live attenuated vaccines (LAVs). Notwithstanding their crucial role in BTV control, these vaccines present several drawbacks, such as their serotype-specificity or the inability to distinguish between vaccinated and infected animals (differentiating infected from vaccinated animals [DIVA] strategy). In this sense, experimental approaches have been implemented with variable success so far. Subunit vaccines, virus-like particle (VLP)-based vaccines, DNA vaccines, recombinant viral vector vaccines, and implementation of reverse genetics systems for generation of new attenuated disabled infectious single animal (DISA) or replication-defective (disabled infectious single cycle [DISC]) vaccines, have targeted proteins located in the outer capsid of the virion, thereupon inducting nAbs against BTV (13–15). In the majority of cases, despite their DIVA character, cross-protection is slightly or not achieved due to the highly variable amino acidic sequence of VP2 among serotypes. The combination of both arms of the adaptive immune response, virus nAbs and cytotoxic T lymphocytes (CTLs), is crucial for the development of a long lasting and protective immunity against BTV in animals (16, 17). The nonstructural protein 1 (NS1), an upregulator of viral protein synthesis (18, 19), is the most expressed viral protein along with NS2 upon infection. In addition, both proteins are highly conserved among BTV serotypes (20), which fosters their utilization as attractive targets for therapeutic antiviral intervention strategies, although both proteins do not induce neutralizing activity (21). What is more, it has been identified the presence of CD8^+^ T cell epitopes within the sequence of NS1 (22, 23) and some works have pointed out the potential of NS2 to induce cellular immune responses against BTV (24–26).

The induction of strong T cell responses against multiple intracellular pathogens has been attained by utilization of viral vaccine vectors, like modified vaccinia virus Ankara (MVA) (13). For BTV, homologous or heterologous prime-boost immunization regimes using MVA expressing NS1 or its truncated form NS1-Nt, either individually or in combination with structural proteins VP2 and VP7, have conferred total protection against heterologous viral challenges in IFNAR(-/-) mice (22, 27, 28). However, homologous prime-boost strategies could induce a limited response due to the development of a specific immune response against the viral vector, diminishing the antigenicity of the second immunization. Oftentimes, recombinant MVA vectors have been assayed as boost in combination with recombinant adenovirus vectors, like chimpanzee adenovirus Oxford 1 (ChAdOx1), procuring significantly increased CD8^+^ T cell responses compared to prime-only strategies (29–31). Replication-defective chimpanzee adenoviruses (ChAd), including ChAdOx1, have been qualified as a safe vaccine platform for a wide range of infectious diseases, including the emerging SARS-CoV-2 (32–37). Besides, this vaccine vector possess a huge potentiality because of their lack of pre-existing anti-vector immunity, thus excluding restrictions in vaccine efficacy (38).

In a previous work, we reported a robust T cell mediated immune response after application of a prime-only with ChAdOx1 and a heterologous prime-boost strategy combining both MVA and ChAdOx1 expressing NS1 in IFNAR(-/-) mice, conferring a full protection against BTV-4M in the short and long term. Moreover, immunization of sheep with ChAdOx1/MVA-NS1 cushioned the rise of fever and prevented the upsurge of viremia (39). Here, MVA and ChAdOx1-vectored vaccines were designed to simultaneously express the immunogenic NS1 protein and/or NS2-Nt, the N-terminal half of protein NS2 (NS2_1-180_). We evaluated the immunogenicity of NS2-Nt and we showed that the combination of NS1 and NS2-Nt increases the protection elicited by NS1 alone in both the murine model and sheep, one of the BTV natural hosts. Additionally, the multiserotype character of the vaccine candidates was confirmed in mice. Finally, studies of humoral and T cell mediated immune responses were conducted in mice and sheep to spot the basis of the conferred protection.

## RESULTS

### Protein NS2 and its N-terminal half induce antigen-specific cellular immune responses.

The nonstructural protein NS2 is one of the most conserved proteins among BTV serotypes (20), making it a good candidate to be studied as an antigen in a multiserotype vaccine against BTV. In fact, the percent identity of the amino acid sequences of the BTV serotypes used in this work is higher than 97% (data not shown). In order to identify theoretical CD8^+^ T-cell epitopes, NS2 sequence was analyzed using the prediction algorithms IEDB and SYFPEITHI and considering the major histocompatibility complex (MHC) haplotype from the mice used in the study. The combination of the best score in the two databases showed the presence of theoretical CD8^+^ T-cell epitopes located in the N-terminal half of the sequence (Table 1).

**TABLE 1:** NS2 peptides identified from the epitope prediction in H-2 Db haplotype

Protein	Initial position	Length	Sequence	Score
IEDB (Immune Epitope Database and Analysis Resource)	265	9	KTHITKEYI	0.21497
	14	9	VLDANAKTL	0.155801
	31	9	SQPYCQIKI	0.154732
	7	9	RFTKNIFVL	0.126527
	107	9	RVQHNGVMV	0.117291
	96	9	FEGVSVTPM	0.085757
	124	9	GMGIVQPYM	0.07383
	71	9	GQDIISLML	0.066846
SYFPEITHY (Database of MHC Ligands and Peptide Motifs)	14	9	VLDANAKATL	24
	7	9	RFTKNIFVL	18
	96	9	FEGVSVTPM	17
	296	9	QASGGFDRM	17
	31	9	SQPYCQIKI	16
	46	9	KPVKNPEPK	16
	111	9	NGVMVDAEI	16
	293	9	AMPQASGGF	15

**FIG 1:**
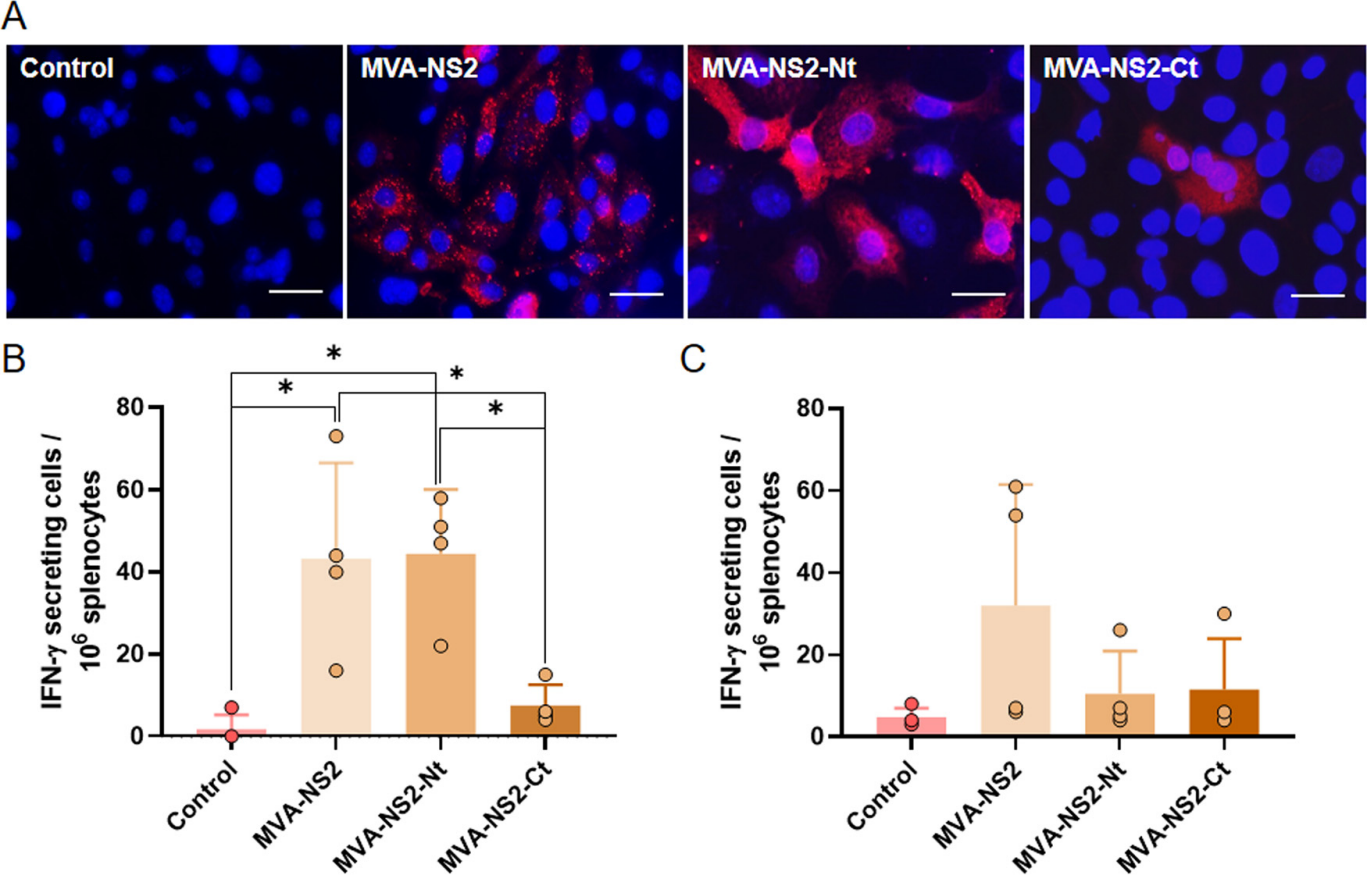
Cellular immune responses against BTV in mice immunized with MVA-NS2, MVA-NS2-Nt, and MVA-NS2-Ct. (A) Indirect immunofluorescence of DF-1 cells infected with MVA-NS2, MVA-NS2-Nt, MVA-NS2-Ct, or noninfected (control). NS2, NS2-Nt, and NS2-Ct (red) were detected using MAb 23H6 α-NS2. Nuclei were stained with DAPI. Scale bars 20 μm. (B, C) Interferon gamma ELISPOT assay. Splenocytes of IFNAR(-/-) mice immunized with MVA-NS2, MVA-NS2-Nt, or MVA-NS2-Ct were stimulated with the recombinant proteins NS2-Nt (B) or NS2-Ct (C). Points represent individual values for each mouse, bars represent the mean values of each group and errors bars represent *SD*. Asterisks denote significant differences between stimulated splenocytes (*P*  <  0.05) (The Mann–Whitney U test).

In the light of the above, we decided to evaluate the cellular immune response elicited by the NS2 BTV protein when it is delivered by the MVA viral vaccine vector in IFNAR(-/-) mice. For this purpose, recombinant MVAs (rMVAs) expressing NS2 or its N- or C-terminal half (amino acids 1 to 180 or 178 to 354, respectively) were generated. The correct expression of NS2, NS2-Nt, or NS2-Ct by these rMVAs was evaluated and confirmed by immunofluorescence assay (IFA) (Fig. 1A).

In order to assess the antigen-specific cellular immune response induced by NS2 or its N- or C-terminal half, we immunized IFNAR(-/-) mice following a homologous prime-boost with MVA-NS2, MVA-NS2-Nt, or MVA-NS2-Ct. Splenocytes were harvested 14 days after boost and a gamma interferon (IFN-γ) enzyme-linked immunosorbent spot (ELISPOT) assay was performed. After restimulation with protein NS2-Nt, splenocytes of mice immunized with MVA-NS2 or MVA-NS2-Nt yielded detectable specific IFN-γ-producing cells (mean spots: 43.25; mean spots: 44.5) (Fig. 1B). The levels of IFN-γ producing cells induced by MVA-NS2-Nt were found significantly higher when compared with control (mean spots: 1.75) and MVA-NS2-Ct (mean spots: 7.5) immunization groups, which failed to induce a specific cellular immune response as expected (Fig. 1B). Besides, restimulation with polypeptide NS2-Ct of splenocytes from mice immunized with MVA-NS2-Ct did not yield a significant response, as the number of specific IFN-γ-producing cells (mean spots: 11.5) was similar to that of the control group (mean spots: 4.75) (Fig. 1C), which support the data of the *in silico* analysis that indicated the prevailing location of CD8^+^ T cell epitopes in the N-terminal half of the protein (Table 1). Overall, these results reveal that the NS2-Nt protein delivered by MVA is able to stimulate a specific cellular immune response in IFNAR(-/-) mice.

### Evaluation of BTV-4 NS1 and NS2-Nt expression from recombinant MVA and ChAdOx1.

Recent data from our laboratory has shown that the cloning of heterologous antigens in the F13L and TK loci of the MVA genome induced a strong immune response against these antigens in immunized animals (40). Following this rationale, we generated rMVAs that express a single BTV gene cloned in the F13L locus (MVA-NS1 or MVA-NS2-Nt) and one rMVA that expresses these two BTV genes cloned in the F13L and TK loci (MVA-NS1-NS2-Nt). Considering the results of the previous section regarding the immunogenicity of NS2-Nt and also contemplating the constraints related with the cargo capacity of ChAdOx1 (< 5 Kb) (38), we decided to formulate the N-terminus half of NS2 in both recombinant MVA and ChAdOx1 (rChAdOx1) viral vectors.

**FIG 2:**
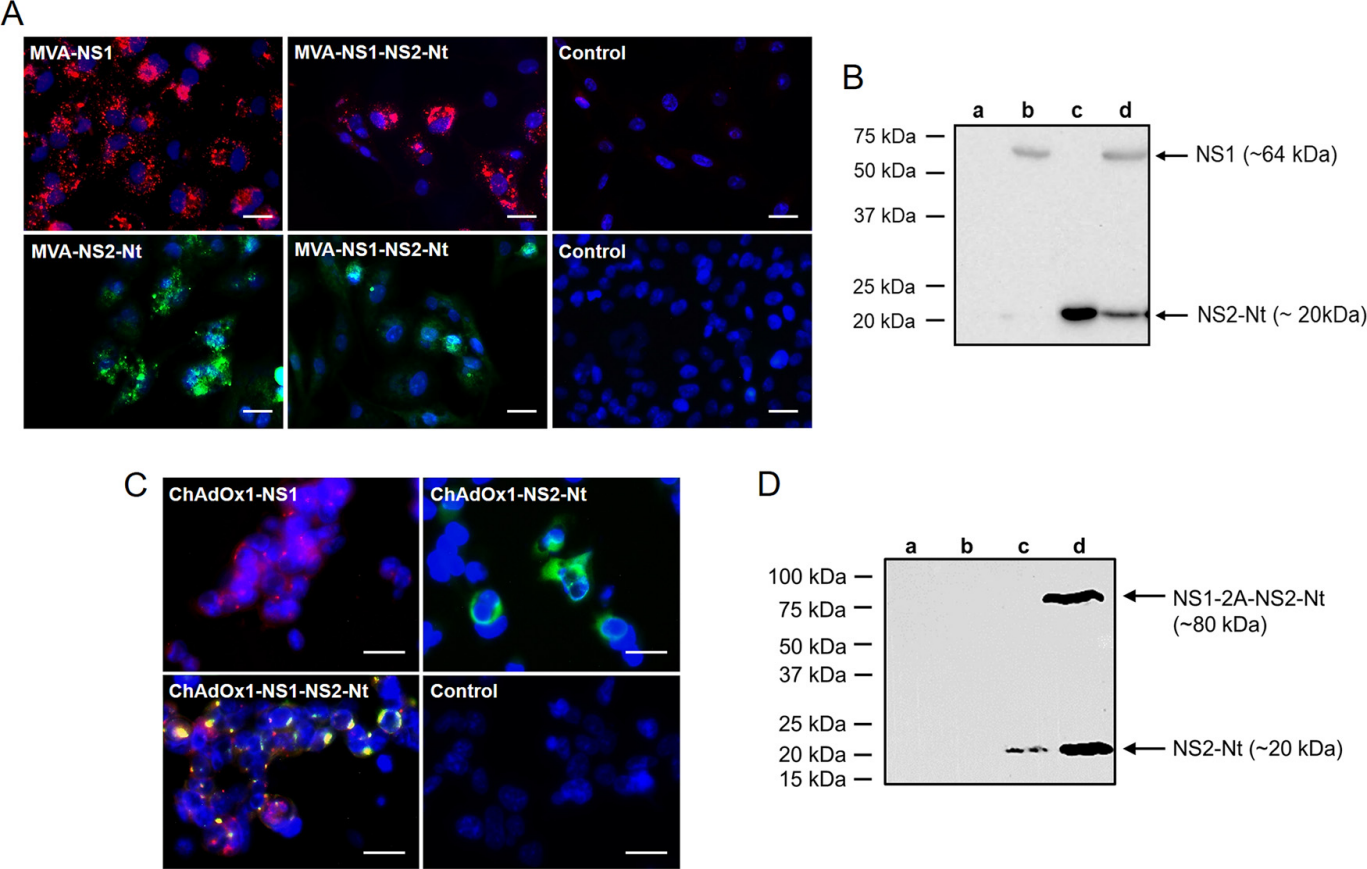
Expression analysis of heterologous BTV proteins by rMVA and rChAdOx1. (A) Indirect immunofluorescence of DF-1 cells infected (MOI  =  1) with MVA-NS1, MVA-NS2-Nt, MVA-NS1-NS2-Nt, or noninfected (control). NS1 protein (red) was detected using a mouse polyclonal hyperimmune serum against ChAdOx1-NS1. NS2-Nt (green) was detected using MAb 23H6 α-NS2. Nuclei were stained with DAPI. Scale bars 20 μm. (B) Immunoblot analysis of noninfected DF-1 cells (lane a) or infected with MVA-NS1 (lane b), MVA-NS2-Nt (lane c) or MVA-NS1-NS2-Nt (lane d) at 18 h.p.i. using a mouse hyperimmune serum against BTV-4. (C) Indirect immunofluorescence of HEK293 cells infected (MOI  =  0.5) with ChAdOx1-NS1, ChAdOx1-NS2-Nt, ChAdOx1-NS1-NS2-Nt, or noninfected (control). NS1 (red) was detected using a sheep polyclonal hyperimmune serum against MVA-NS1. NS2-Nt was detected using MAb 23H6 specific of NS2-Nt. Nuclei were stained with DAPI. Scale bars 50 μm. (D) Immunoblot analysis of HEK293 cells noninfected (lane a) or infected with ChAdOx1-NS1 (lane b), ChAdOx1-NS2-Nt (lane c) or ChAdOx1-NS1-NS2-Nt (lane d) at 18 h.p.i. using a MAb 23H6 α-NS2. Numbers indicate relative molecular mass in Kilodaltons (kDa).

The correct expression of the heterologous BTV antigens cloned in the recombinant MVA and ChAdOx1 was confirmed by IFA and immunoblot. DF-1 cells were infected with the rMVAs and the characteristic spotted pattern of NS1 was observed in DF-1 cells infected with MVA-NS1 and MVA-NS1-NS2-Nt (Fig. 2A). Expression of NS2-Nt by rMVAs was also revealed in infected DF-1 cells (Fig. 2A). Noninfected cells did not show evidences of a specific signal of NS1 or NS2-Nt in any case. Immunoblotting also showed expression of the heterologous BTV antigens by rMVAs in DF-1-infected cells, with the use of a sheep hyperimmune serum against BTV that enabled detection of the presence of both NS1 and NS2-Nt proteins with the expected molecular mass (Fig. 2B).

Similarly, IFA was performed in HEK293 cells infected with rChAdOx1 expressing NS1 and/or NS2-Nt to assess the appropriate expression of these BTV antigens. Expression of NS1 and NS2-Nt proteins was observed with the characteristic spotted pattern of NS1 in infected HEK293 cells (Fig. 2C). NS1 and NS2-Nt proteins were cloned in the rChAdOx1 as a fusion protein including the foot-and-mouth disease virus (FMDV) 2A “ribosomal skipping” linker (2A) into the fusing point. Colocalization of NS1 and NS2-Nt was exhibited by cells infected with ChAdOx1-NS1-NS2-Nt. This overlapping signal may be caused by the expression of the polyprotein NS1-2A-NS2-Nt although it cannot be ruled out a likely interaction between NS1 and NS2-Nt. To confirm the expression and “cleavage” of the NS1-2A-NS2-Nt polyprotein, the infected cell extracts were analyzed by immunoblot at 18-h postinfection (h.p.i.). The monoclonal Ab 23H6 specific of NS2 allowed the detection of the fused NS1-2A-NS2-Nt insert and the protein NS2-Nt (Fig. 2D, lane d) with the expected molecular masses, confirming the presence of both cleaved and uncleaved forms of the polyprotein NS1-2A-NS2-Nt in the infected cells.

Altogether, these data confirm the efficient expression of these proteins from BTV-4 cloned in the rMVA and rChAdOx1 vaccine vectors to be used for immunization assays in IFNAR(-/-) mice and sheep.

### The combined expression of NS1 and NS2-Nt of BTV improves the protection conferred against BTV-4M in IFNAR(-/-) mice.

Considering the immunogenicity of NS2-Nt, we decided to assess the potentiality to confer protection against BTV of NS2-Nt protein, individually or combined with NS1. Adult IFNAR(-/-) mice were immunized with a single dose of rMVA (MVA-NS1, MVA-NS2-Nt, or MVA-NS1-NS2-Nt) or rChAdOx1 (ChAdOx1-NS1, ChAdOx1-NS2-Nt, or ChAdOx1-NS1-NS2-Nt) by intraperitoneal or intramuscular route, respectively. Two (rMVA) or four (rChAdOx1) weeks after immunization, mice were subcutaneously challenged with a lethal dose of BTV-4M. Survival and viremia were subsequently analyzed.

**FIG 3:**
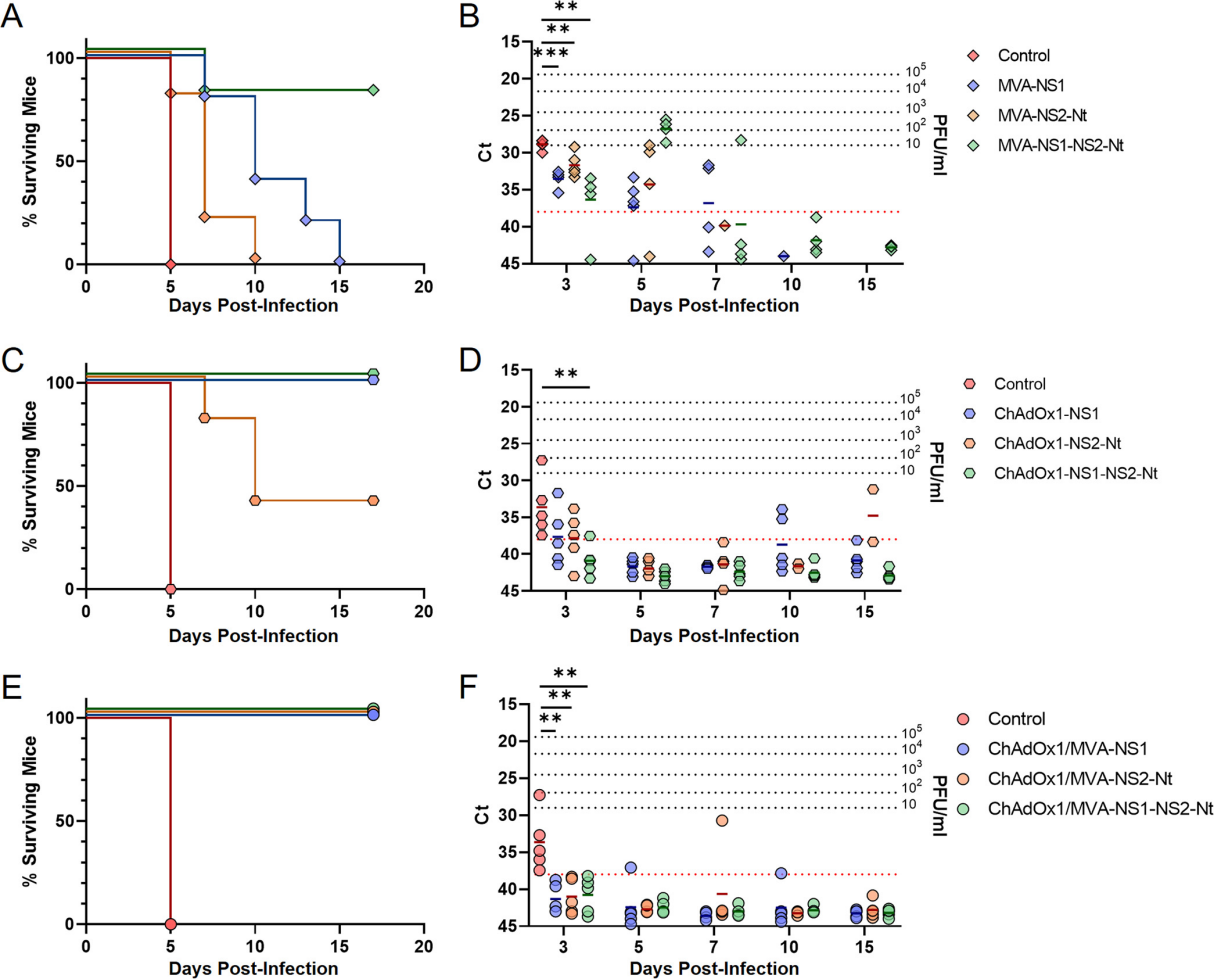
Protection of immunized IFNAR(-/-) mice against a lethal challenge with BTV-4M. Groups of IFNAR(-/-) mice (*n*  =  5) were immunized with (A, B) a single dose of MVA-NS1, MVA-NS2-Nt, or MVA-NS1-NS2-Nt; (C, D) a single dose of ChAdOx1-NS1, ChAdOx1-NS2-Nt, or ChAdOx1-NS1-NS2-Nt; or (E, F) following a heterologous prime-boost regimen consisting of an initial dose of rChAdOx1 (prime) followed by a second dose of rMVA (boost). In all cases, a group was left untreated (control). Immunized and nonimmunized mice were challenged with a lethal dose of BTV-4M. (A, C, E) Survival rates after infection. Curves were found statistically significant compared with nonimmunized survival curve as calculated by Log-rank test (*P value*  <  0.05). (B, D, F) Viremia analyzed by RT-qPCR of nonimmunized and immunized IFNAR(-/-) mice after viral challenge. Expression of mRNA of segment 5 (encoding NS1 protein) was quantified at 3, 5, 7, 10, and 15 d.p.i. Results were expressed as Ct (left *y* axis) and PFU/mL equivalents (right *y* axis and dotted horizontal lines). The real-time RT-qPCR specific for BTV segment 5 was performed as described by Toussaint et al. (83) and mouse blood containing different concentrations of virus were titrated and used as standards (22, 39). Cut-off Ct  ≥  38 (dotted pink line). Points represent individual Ct for each mouse and lines of the corresponding color represent the mean Ct value of each group. Differences between groups were calculated by multiple *t* test analysis using the Sidak–Bonferroni method. ** *P value*  <  0.002; *** *P value*  <  0.001.

Mice immunized with MVA-NS2-Nt (Fig. 3A) experimented a delay in appearance of clinical signs and death compared with the control group, which succumbed to infection with BTV-4M at day 5 postchallenge, in contrast to immunized mice with NS2-Nt that died after 10 days postinfection (d.p.i.). A similar pattern was observed for those mice immunized with MVA-NS1 although these animals died between 7 and 15 d.p.i. (Fig. 3A). Interestingly, 80% of the mice immunized with MVA-NS1-NS2-Nt survived to the infection with BTV-4M (Fig. 3A). All animals immunized with the different rMVAs showed similar levels of viremia at 3 d.p.i., being significantly lower compared with nonimmunized mice (Ct value mean: 28.868). Nevertheless, although mice immunized with MVA-NS1, MVA-NS2-Nt, and MVA-NS1-NS2-Nt (Fig. 3B) were viremic at 5 d.p.i., viremia was completely abrogated in subsequent days in mice immunized with MVA-NS1-NS2-Nt, the only group of animals that survived the challenge with BTV-4M, reaching the aviremic status (Ct value  ≥ 38) at 10 d.p.i. (Ct value mean: 42.807).

A similar outcome was observed after immunization with rChAdOx1. IFNAR(-/-) mice immunized with a single dose of ChAdOx1-NS1 survived to infection (Fig. 3C). These animals were viremic after infection but the presence of virus in blood was undetectable at day 15 postinfection (Ct value mean: 40.86) (Fig. 3D). Animals immunized with a single dose of ChAdOx1-NS2-Nt were viremic (Ct value mean: 34.755) and 60% of them succumbed to the infection at 15 d.p.i. (Fig. 3C and D). In contrast, all mice immunized with the rChAdOx1 simultaneously expressing NS1 and NS2-Nt survived to infection with BTV-4M (Fig. 3C), showing an aviremic status (Ct value  ≥ 38) throughout the experiment (Fig. 3D).

Thereafter, we aimed to evaluate the protective efficacy of a prime-boost strategy combining both recombinant viral vectors ChAdOx1 and MVA expressing NS1 and/or NS2-Nt. We applied a heterologous prime-boost regimen with rChAdOx1 (ChAdOx1-NS1, ChAdOx1-NS2-Nt, or ChAdOx1-NS1-NS2-Nt) followed by a dose of rMVA (MVA-NS1, MVA-NS2-Nt, or MVA-NS1-NS2-Nt) in IFNAR(-/-) mice 4 weeks apart. Two weeks after immunization with rMVAs, mice were challenged with a lethal dose of BTV-4M, and survival and viremia were analyzed. All control mice died by day 5 postinfection. In contrast, none of the mice belonging to ChAdOx1/MVA-NS1 and ChAdOx1/MVA-NS2-Nt immunization groups died after infection (Fig. 3E). Some of these animals displayed low but detectable levels of viremia, although they became aviremic (Ct value  ≥ 38) at day 15 postinfection (Fig. 3F). In addition, all mice immunized with ChAdOx1/MVA-NS1-NS2-Nt neither succumbed to infection (Fig. 3E) nor showed viremia at any day after challenge (Fig. 3F).

These data mostly indicate that the joint expression of NS1 and NS2-Nt by either rMVA or rChAdOx1 optimizes the protection elicited by NS1 against BTV-4M. Moreover, the prime-boost strategy turned out to be more efficacious in protection against BTV infection than prime-only strategies in IFNAR(-/-) mice.

### ChAdOx1/MVA-NS1-NS2-Nt provides total multiserotype protection in IFNAR(-/-) mice.

**FIG 4:**
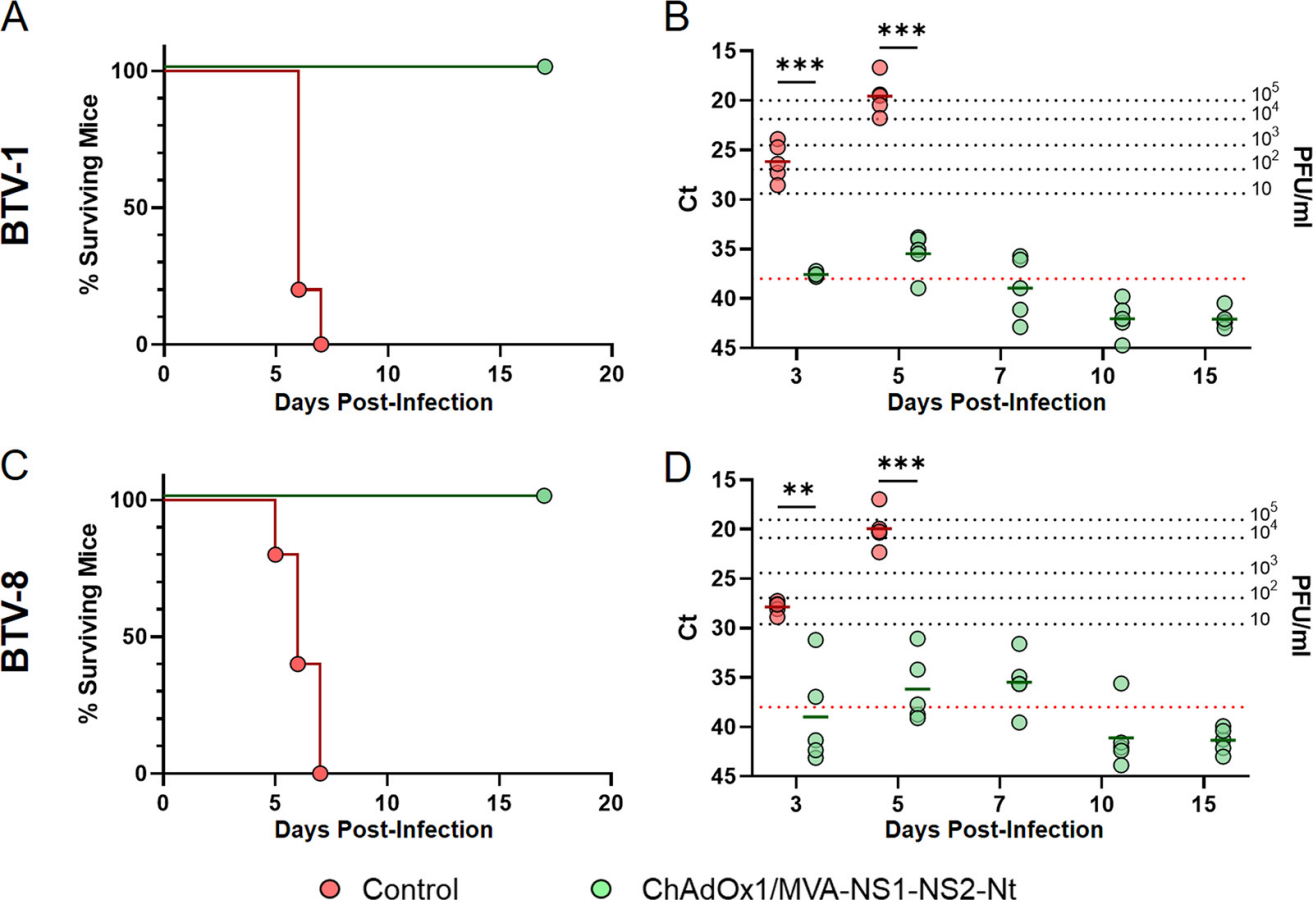
Protection elicited by ChAdOx1/MVA-NS1-NS2-Nt in IFNAR(-/-) mice against a lethal challenge with BTV-1 and BTV-8. Groups of IFNAR(-/-) mice (*n*  =  5) were immunized following a heterologous prime-boost regimen consisting of an initial dose of ChAdOx1-NS1-NS2-Nt followed by a second dose of MVA-NS1-NS2-Nt. A group was left untreated (control). Immunized and nonimmunized mice were challenged with a lethal dose of BTV-1 or BTV-8. (A, C) Survival rates after infection. Curves were found statistically significant compared with nonimmunized survival curve as calculated by Log-rank test (*P value*  <  0.05). (B, D) Detection of BTV-1 and BTV-8 by RT-qPCR in blood of nonimmunized and immunized IFNAR(-/-) mice after viral challenge. Expression of mRNA of segment 5 (encoding NS1 protein) was quantified at 3, 5, 7, 10, and 15 d.p.i. Results were expressed as Ct (left *y* axis) and PFU/mL equivalents (right *y* axis and dotted horizontal lines). The real-time RT-qPCR specific for BTV segment 5 was performed as described by Toussaint et al. (83) and mouse blood containing different concentrations of virus were titrated and used as standards (22, 39). Cut-off Ct  ≥  38 (dotted pink line). Points represent individual Ct for each mouse and lines of the corresponding color represent the mean Ct value of each group. Differences between groups were calculated by multiple *t* test analysis using the Sidak–Bonferroni method. ** *P value*  <  0.002; *** *P value*  <  0.001.

In preceding works, we confirmed that homologous prime-boost immunization with MVA-NS1 or heterologous prime-boost immunization with ChAdOx1/MVA-NS1 provides protection against several BTV serotypes (22, 39). With a view to prove the multiserotype character of the prime-boost ChAdOx1/MVA-NS1-NS2-Nt, groups of IFNAR(-/-) mice were immunized and challenged 2 weeks postboost with a lethal dose of BTV-1 or BTV-8 (Fig. 4).

By day 7 postchallenge, all control mice had succumbed to infection with either BTV-1 or BTV-8 (Fig. 4A and C). In both cases, these nonimmunized animals were viremic from day 3 postinfection, with the highest Ct values at 5 d.p.i. (Fig. 4B and D). Contrariwise, mice immunized with ChAdOx1/MVA-NS1-NS2-Nt were fully protected from viral infection with BTV-1 or BTV-8. Indeed, after challenge with BTV-1, immunized mice displayed significantly lower and almost undetectable levels of viremia at 3 and 5 d.p.i. (Fig. 4B), with mean Ct values of 37.56 and 35.45, respectively, followed by a reduction in their levels of viremia from then on. Similarly, immunized mice challenged with BTV-8 also displayed some detectable levels of viremia at day 3 (Ct value mean: 38.99), day 5 (Ct value mean: 36.16), and day 7 (Ct value mean  =  35.472) p.i., but significantly lower compared with those of the control group in any case, and no virus was detected in blood of these immunized mice from 10 d.p.i. (Fig. 4D). All these data confirm the capability of this vaccine candidate to protect against different BTV serotypes in the murine model.

### The multiserotype protection elicited by the heterologous ChAdOx1/MVA immunization largely depends on a BTV-specific cellular immune response.

**FIG 5:**
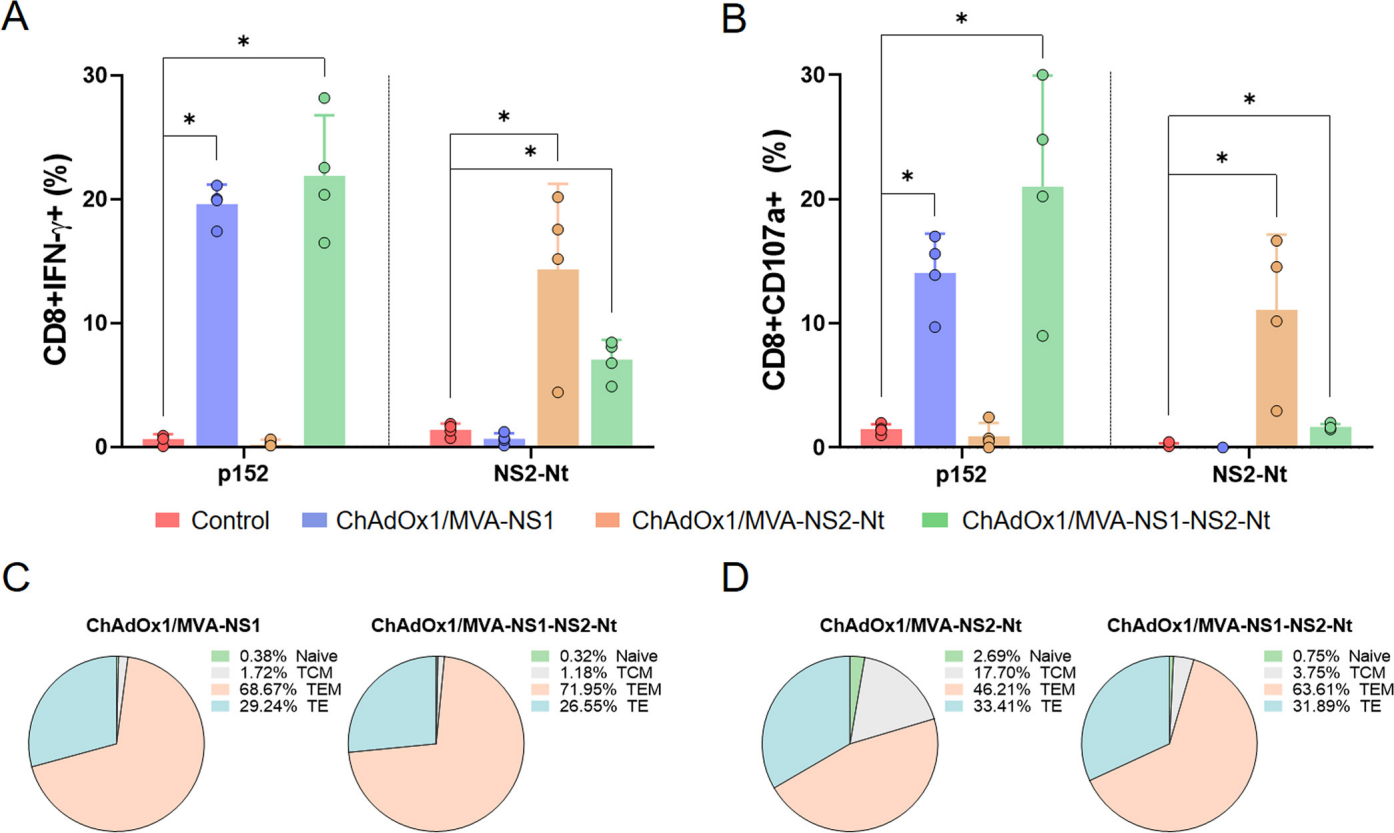
Cellular immune responses against BTV in ChAdOx1/MVA immunized mice. Percentage of CD8+IFN-γ+T cells (A) and CD8+CD107a+ T cells (B) after stimulation with peptide 152 (NS1) and NS2-Nt. Points represent individual values for each mouse, bars represent the mean values of each group and error bars represent *SD*. Asterisks denote significant differences between immunized and control mice (*P*  <  0.05) (The Mann–Whitney U test). * *P value*  <  0.05. (C, D) Pie charts represents the phenotypic profile of BTV-specific CD8^+^ T cells of immunized mice. CD127 and CD62L expression was used to identify naive, TCM, TEM, and TE subpopulations. Each slice corresponds to the proportion of each BTV-specific CD8^+^ T cell subpopulations within the total NS1 (C) or NS2-Nt (D) specific CD8^+^ T cells producing IFN-γ.

Lately, we demonstrated that the multiserotype protection promoted by NS1 is built upon the induction of CTLs (22, 39). Conversely, the importance of the nonstructural protein NS2 in CD8^+^ T cell-mediated protection against BTV needs to be further characterized, although NS2-specific lymphocyte proliferative responses have been described (24). In consequence, Intracellular Cytokine Staining (ICS) was performed to analyze the cellular immune response elicited by the heterologous prime-boost regime ChAdOx1/MVA expressing both NS1 and NS2-Nt (Fig. 5).

To evaluate the capability of this prime-boost to induce a cellular immune response, we measured IFN-γ production as well as CD107a cytotoxic expression marker in CD8^+^ T cells by ICS after restimulation of splenocytes from immunized and nonimmunized mice with the NS1 immunodominant peptide p152 (9-mer peptide GQIVNPTFI) or the purified recombinant protein NS2-Nt. The peptide p152 induced high and significant levels of CD8+IFN-γ+T cells as well as CD8+CD107a+ T cells after restimulation of splenocytes from animals immunized with ChAdOx1/MVA-NS1 or ChAdOx1/MVA-NS1-NS2-Nt (Fig. 5A and B). Similarly, splenocytes of mice immunized with ChAdOx1/MVA-NS2-Nt or ChAdOx1/MVA-NS1-NS2-Nt displayed significant levels of CD8+IFN-γ+ and CD8+CD107a+ T cells upon restimulation with NS2-Nt recombinant protein compared with nonimmunized animals, although the levels of the ChAdOx1/MVA-NS1-NS2-Nt immunized mice were lower than those from the ChAdOx1/MVA-NS2-Nt immunization group. In any case, the nonimmunized group did not display a perceivable response to these stimuli.

Moreover, we also determined the phenotype of the BTV-specific CD8^+^ T cells by evaluating the presence of CD127 and CD62L surface markers, which define memory subpopulations such as T central memory (TCM; CD127^+^/CD62L^+^), T effector memory (TEM; CD127^+^/CD62L^−^), and T effector (TE; CD127^−^/CD62L^−^) T cells. Restimulation with p152 predominantly led to high levels of TEM and TE in groups immunized with ChAdOx1/MVA-NS1 (68.67% and 29.24%, respectively) and ChAdOx1/MVA-NS1-NS2-Nt (71.95% and 26.55%, respectively) (Fig. 5C). Likewise, a predominant TEM and TE response was observed after NS2-Nt restimulation in ChAdOx1/MVA-NS2-Nt (46.21% and 33.41%, respectively) and ChAdOx1/MVA-NS1-NS2-Nt immunization groups (63.61% and 31.89%, respectively) (Fig. 5D). However, restimulation with NS2-Nt of groups immunized with ChAdOx1/MVA-NS2-Nt and ChAdOx1/MVA-NS1-NS2-Nt led to a more pronounced TCM response, specially the group immunized with ChAdOx1/MVA-NS2-Nt (17.70%), whereas the induced TCM phenotype was residual after NS1 restimulation, which highlights differences between the phenotype of the CD8^+^ T memory subpopulations induced by NS1 or NS2-Nt.

**FIG 6:**
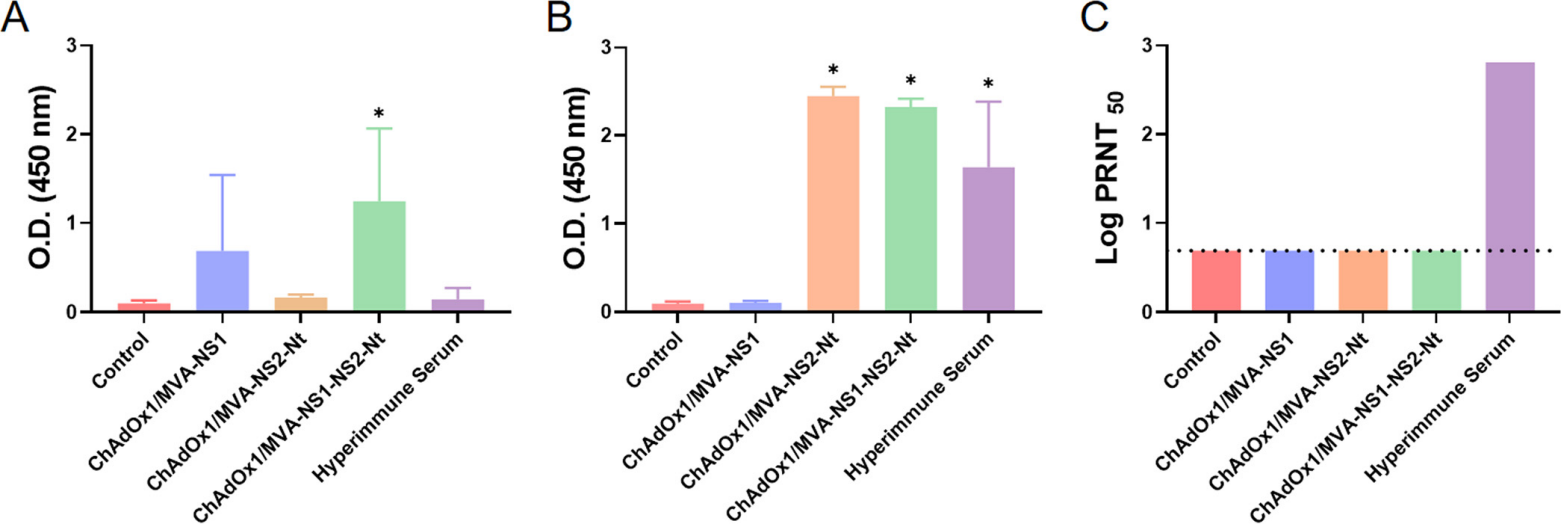
Humoral responses against BTV elicited by MVA and ChAdOx1 in IFNAR(-/-) mice. (A, B) Induction of IgG NS1 and NS2 antibodies, respectively, by indirect ELISA in vaccinated animals. Sera dilution 1:50. (C) Neutralizing antibodies titers against BTV in immunized animals by plaque reduction neutralization assay. Cut-off: 0.69 (log 5). Hyperimmune serum: serum from mice infected with BTV-4M. Results were expressed as optical densities (ODs) measured at 450 nm. Bars represent the mean values of each group and error bars represent *SD*. Asterisks denote significant differences between immunized and control mice (*P*  <  0.05) (The Mann–Whitney U test). * *P value*  <  0.05.

Virus neutralization tests (VNT) and enzyme-linked immunosorbent assays (ELISA) specifically for NS1 and NS2-Nt antibodies were also conducted to analyze the humoral immune response induced by this heterologous prime-boost strategy. The presence of specific antibodies to NS1 or NS2-Nt was analyzed 2 weeks postboost. Levels of antibodies specific to NS1 were not significant in all cases except for those mice immunized with ChAdOx1/MVA-NS1-NS2-Nt (*P*  =  0.0285; mean optical density at 450 nm (OD_450_) = 1.449) (Fig. 6A). In the case of antibodies specific to NS2-Nt, mice immunized with ChAdOx1/MVA-NS2-Nt (*P*  =  0.0285; mean OD_450_ = 2.478) or ChAdOx1/MVA-NS1-NS2-Nt (*P*  =  0.0285; mean OD_450_ = 2.310) showed significantly higher levels of antibodies compared with the control group (nonimmunized) (Fig. 6B). As expected, none of the individual sera analyzed showed neutralizing activity against BTV-4M as determined by VNT assay (Fig. 6C), which upholds that the protection observed in mice is based on a protective cytotoxic CD8^+^ T cell response.

### ChAdOx1/MVA-NS1-NS2-Nt confers protection in sheep upon BTV-4M challenge.

**FIG 7:**
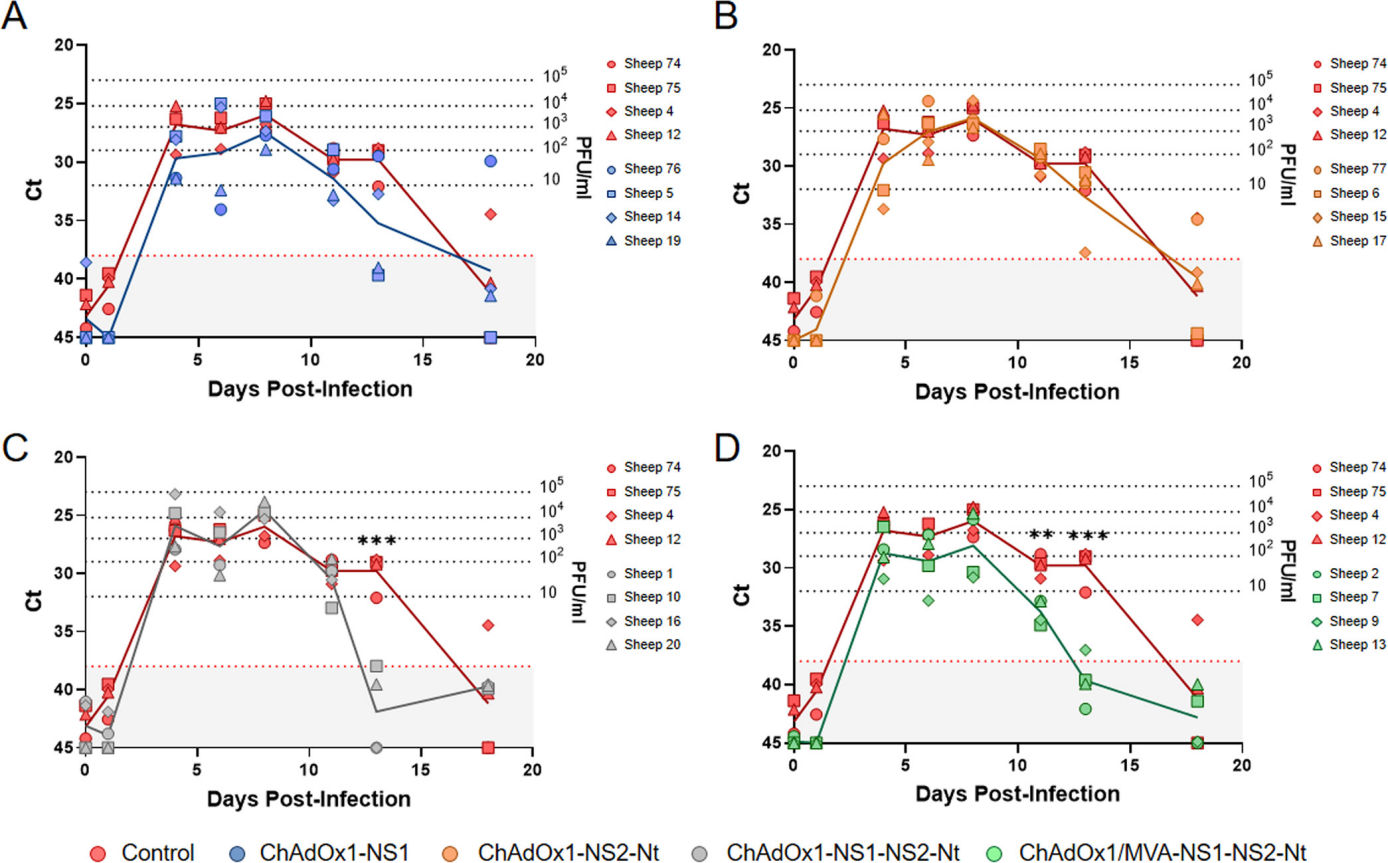
Levels of viremia in immunized sheep after challenge with BTV-4M. Groups of sheep (*n*  =  4) were immunized with a single dose of (A) ChAdOx1-NS1, (B) ChAdOx1-NS2-Nt, (C) ChAdOx1-NS1-NS2-Nt, or (D) following a heterologous prime-boost regimen consisting of a prime dose of ChAdOx1-NS1-NS2-Nt followed by a boost of MVA-NS1-NS2-Nt. A group was left untreated (control). Immunized and nonimmunized sheep were challenged with BTV-4M. Detection of BTV-4M was performed by RT-qPCR in blood of nonimmunized and immunized sheep after viral challenge. Expression of mRNA of segment 5 (encoding NS1 protein) was quantified at 0, 1, 4, 6, 8, 11, 13, and 18 d.p.i. Results were expressed as Ct (left *y* axis) and PFU/mL equivalents (right *y* axis and dotted horizontal lines). The real-time RT-qPCR specific for BTV segment 5 was performed as described by Toussaint et al. (83) and sheep blood containing different concentrations of virus were titrated and used as standards. Cut-off Ct  ≥  38 (dotted pink line). Points represent individual Ct for each sheep and lines represent the mean values of each group. Differences between groups were calculated by multiple *t* test analysis using the Sidak–Bonferroni method. ** *P value*  <  0.002; *** *P value*  <  0.001.

Data on protection in the mouse model encouraged us to evaluate the protective efficacy of different immunization strategies in sheep, one of the most affected BTV natural hosts. To do so, we immunized sheep with a single dose of rChAdOx1 (ChAdOx1-NS1, ChAdOx1-NS2-Nt, or ChAdOx1-NS1-NS2-Nt) (Fig. 7A to C) or a prime-boost strategy ChAdOx1/MVA-NS1-NS2-Nt (Fig. 6D) in a 4-week interval. Six (prime-boost) or 8 (prime-only) weeks after last immunization, sheep were subcutaneously challenged with 10^5^ PFU of BTV-4M strain (isolated from sheep blood in KC insect cells and not previously passed through mammalian cell lines, retaining its high virulence in sheep). All control and immunized sheep displayed a steep rise in their levels of viremia between days 1 and 4 after challenge. Immunization with a single dose of ChAdOx1-NS2-Nt led to an analogous viremia profile to that of the control group, although one immunized sheep (sheep 15) was nearly aviremic (Ct value  =  37.43) at 13 d.p.i. (Fig. 7B). In contrast, immunization with ChAdOx1-NS1 provided a better protection compared to ChAdOx1-NS2-Nt, as sheep 76 and 19 displayed lower viremia levels between days 4 and 6 postinfection, and two out of four immunized sheep (sheep 5 and 19) were aviremic (Ct value  =  39.66; Ct value  =  39.01) from 13 d.p.i. (Fig. 7A), meanwhile three control sheep remained viremic at day 13 postchallenge and even sheep 4 was viremic at 18 d.p.i. In any case, differences of viremia levels observed between ChAdOx1-NS1 or ChAdOx1-NS2-Nt immunization group and nonimmunized sheep were nonsignificant. Combination of NS1 and NS2-Nt resulted in more prominent results. Despite ChAdOx1-NS1-NS2-Nt immunized sheep showed very similar viremia levels to those of the nonimmunized group until 11 d.p.i., immunization with a single dose of ChAdOx1-NS1-NS2-Nt led to significantly (*P value*  <  0.001) lower viremia levels compared with the nonimmunized control group and the aviremic status of all immunized sheep at day 13 postinfection (Ct value mean  =  41.8775) (Fig. 7C). Remarkably, the heterologous prime-boost ChAdOx1/MVA-NS1-NS2-Nt cushioned the early rise of viremia in sheep 9 as well as sheep 7 exhibited an intense reduction from day 4 postinfection. Moreover, despite sheep 2 and 13 presented a viremia profile identical to that of the control group until day 8 postinfection, four out of four sheep subjected to the prime-boost ChAdOx1/MVA-NS1-NS2-Nt displayed significantly (*P value*  <  0.002) lower viremia levels at 11 d.p.i. (Ct value mean  =  33.8825) compared with the control group (Ct value mean  =  29.7725), reaching the aviremic status (*P value*  <  0.001; Ct value mean  =  39.6575) at day 13 postinfection (Fig. 7D). These data indicate that the immunization with ChAdOx1 expressing the combination of NS1 and NS2-Nt reduces the period of viremia after challenge with BTV-4M. Furthermore, the heterologous prime-boost with ChAdOx1/MVA-NS1-NS2-Nt improves the protection in sheep, significantly reducing the level and period of viremia in immunized animals after BTV challenge.

Regarding the evolution of temperatures and clinical signs, the animals in the nonimmunized control group showed an increase in temperature from 5 d.p.i. that lasted around 5 days. All animals reached temperatures above 40.5°C, with peaks up to 41.5°C (sheep 4). From 4 d.p.i., animals also displayed a progressive onset of mild clinical signs (conjunctivitis with mild serous ocular discharge, seromucous nasal discharge and subcutaneous edema on lips). Clinical signs became more severe as disease progressed and peaked between 8 and 9 d.p.i., also appearing other clinical signs such as apathy, reluctance to move, hyperaemia and swelling of the coronary band at the top of the hooves, hyperaemia in gums, cyanotic oral mucosa, subcutaneous edema in submandibular areas, respiratory rales, and cough. Sheep 4 and 75 displayed the highest clinical scores. Clinical signs progressively disappeared and temperatures decreased until reaching normal values in all animals from 11 d.p.i. onwards. In the group of sheep immunized with single dose of ChAdOx1-NS1, three animals displayed an increase in temperature from 4 d.p.i. that lasted between 5 and 8 days, reaching temperatures above 40.5°C with peaks up to 41.8°C (sheep 5), while in one animal (sheep 76) rectal temperature was not increased after challenge. Apart from sheep 76, clinical scores increased in the other animals from 8 d.p.i. Clinical signs disappeared progressively from 12 d.p.i. onwards at the time that temperatures reached normal values. After challenge, animals immunized with a single dose of ChAdOx1-NS2-Nt displayed a progression of temperatures and clinical signs similar to that described in the control group. Rectal temperatures increased from 5 d.p.i. High temperatures lasted between 4 and 6 days and returned to normal values from 11 d.p.i. onwards. On the other hand, clinical scores increased from 8 d.p.i. Only occasional mild clinical signs were recorded at the time that temperatures returned to normal values. In the group of animals immunized with one dose of ChAdOx1-NS1-NS2-Nt, all animals showed an initial increase in temperature between 4 and 5 d.p.i. with values of 40.8°C or higher (up to 41.8°C and 41.3°C in sheep 16 and 10, respectively). After that, temperatures of 40.5°C or higher were recorded for 4 days in a row in three animals (sheep 1, 10, and 16). In such animals, temperatures returning lasted to normal values from 11 d.p.i. In sheep 16, clinical signs were remarkable from 4 to 5 d.p.i. highlighting the presence of conjunctivitis, ocular and nasal discharge, hyperaemia and cyanosis in gums, subcutaneous edema in submandibular areas, and difficulty breathing and respiratory rales, clinical signs that persisted until 10 d.p.i. In sheep 1 and 10, clinical scores similar to those described in sheep 16 were increased from 8 d.p.i., persisting until 11 d.p.i. In sheep 20, apart from a mild increase of clinical scores at 6 d.p.i., clinical signs observed were mild. Finally, in the group of sheep immunized (prime-boost) with ChAdOx1/MVA-NS1-NS2-Nt, temperatures were increased in some animals (sheep 7 and 9) from 4 d.p.i. At day 5 postinfection all animals showed temperatures of 41.2°C or higher (up to 41.9°C in sheep 9). After the initial increase, temperatures of 40.8°C or higher were recorded in all animals for a shorter period than the other groups returning later to normal values. Temperatures decreased until reached normal values in all sheep at 11 d.p.i. This shorter period of hyperthermia coincides with the shorter period of viremia observed in this group of animals compare with the previous ones. Along with mild clinical signs such as conjunctivitis, nasal discharge, hyperaemia in gums, and subcutaneous edema on lips, between 5 and 7 d.p.i. sheep 9, 7, and 2 started displaying moderate respiratory clinical signs characterized by cough, difficult breathing, and respiratory rales which lasted between 3 days and disappeared progressively, so that all animals were completely recovered by 11 d.p.i.

**FIG 8:**
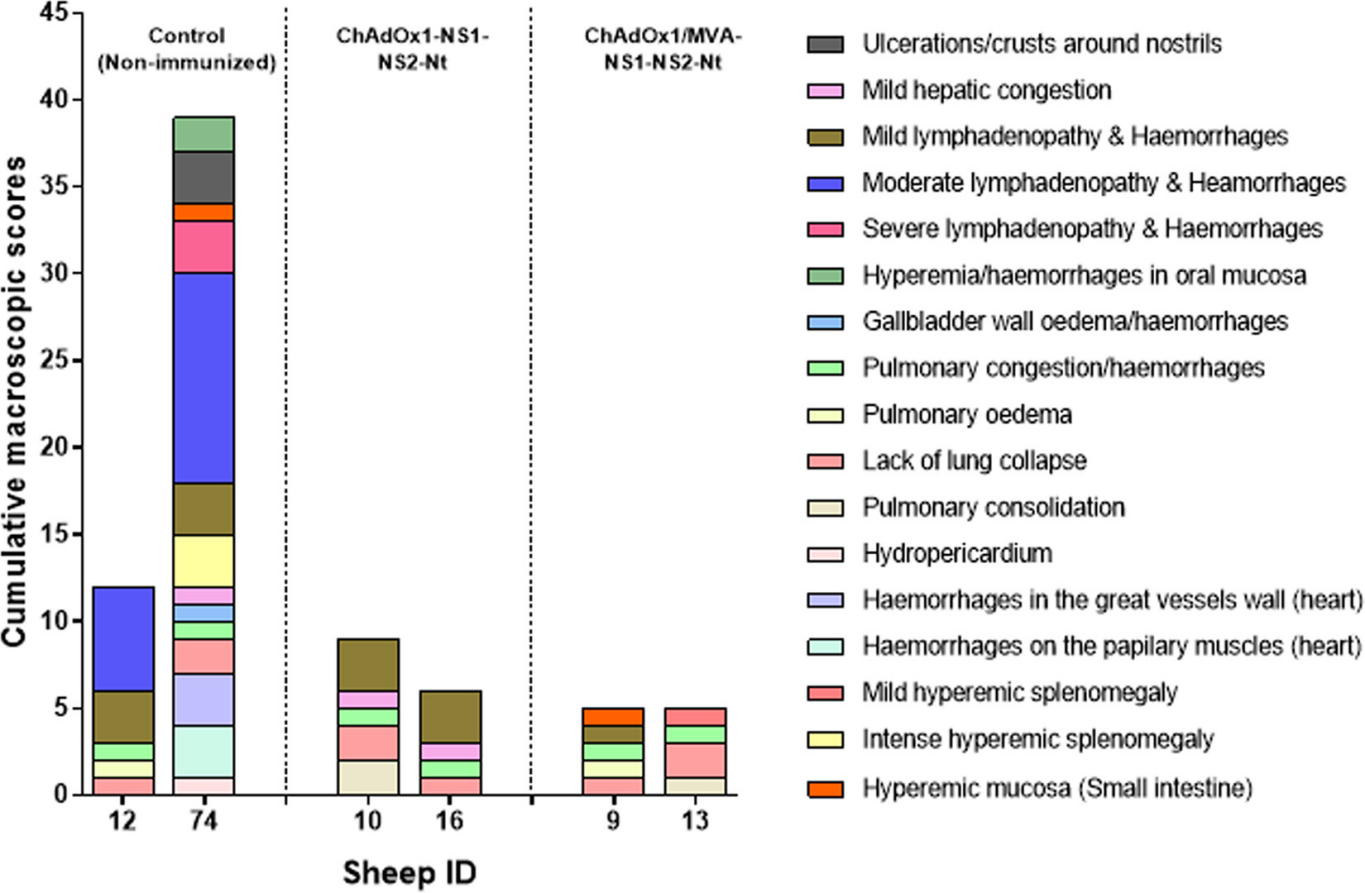
Macroscopic lesions score evaluated in nonimmunized control group as well as in immunized sheep (ChAdOx1-NS1-NS2-Nt and ChAdOx1/MVA-NS1-NS2-Nt immunization groups) after challenge with BTV-4M. Two out of four sheep of the control (sheep 12 and 74) and ChAdOx1-NS1-NS2-Nt (sheep 10 and 16) and ChAdOx1/MVA-NS1-NS2-Nt (sheep 9 and 13) were euthanized at the end of the experiment (18 d.p.i.) and a macroscopic evaluation was conducted during necropsies of the bodies. Sheep ID are represented in the *x* axis. Bars represent the total score of macroscopic lesions for each sheep. Legend information compiles the characteristic BTV lesions evaluated.

To assess the protection conferred by a single immunization with ChAdOx1-NS1-NS2-Nt or a heterologous prime-boost with ChAdOx1/MVA-NS1-NS2-Nt against BTV, two animals per group were euthanized at the end of the experiment and gross lesions were analyzed by postmortem evaluation. During the necropsies carried out at day 18 postinfection, only animals within the nonimmunized control group displayed remarkable macroscopic lesions, some of them characteristic of BTV infection. Macroscopic lesion scores were especially high in the nonimmunized sheep 74 (Fig. 8). This animal showed crusty exudates around the nostrils, hyperemia of oral mucosa and gums, mild hydropericardium, petechial hemorrhages on the papillary muscles of the left ventricle, sub-intimal petechial hemorrhages in the pulmonary artery, mild pulmonary congestion with occasional petechiae, mild congestion in liver along with mild edema in gallbladder wall, and severe hyperemic splenomegaly with marked bleeding after sectioning. Most of the lymph nodes (LNs) evaluated displayed mild to moderate lymphadenopathy in some cases (prescapular, axillary, and submandibular LNs), accompanied by the presence of hemorrhages in cortex and medulla. Regarding sheep 12, macroscopic score was lower and lesions were occasional, less severe, and unspecific (mild pulmonary congestion and edema, mild lymphadenopathy with occasional petechial hemorrhages mainly affecting prescapular and submandibular LNs). Sheep belonging to the aforementioned immunized groups (ChAdOx1-NS1-NS2-Nt and ChAdOx1/MVA-NS1-NS2-Nt) displayed scarce, mild, and unspecific macroscopic lesions, such as mild pulmonary congestion, mild hepatic congestion, mild lymphadenopathy without hemorrhages, and mild hyperemic splenomegaly. Some animals (sheep 10 and 13) also displayed small areas of pulmonary consolidation (chronic pneumonia) in apical areas of cranial pulmonary lobes, although these chronic lesions were associated to undetermined secondary pulmonary pathogens.

**FIG 9:**
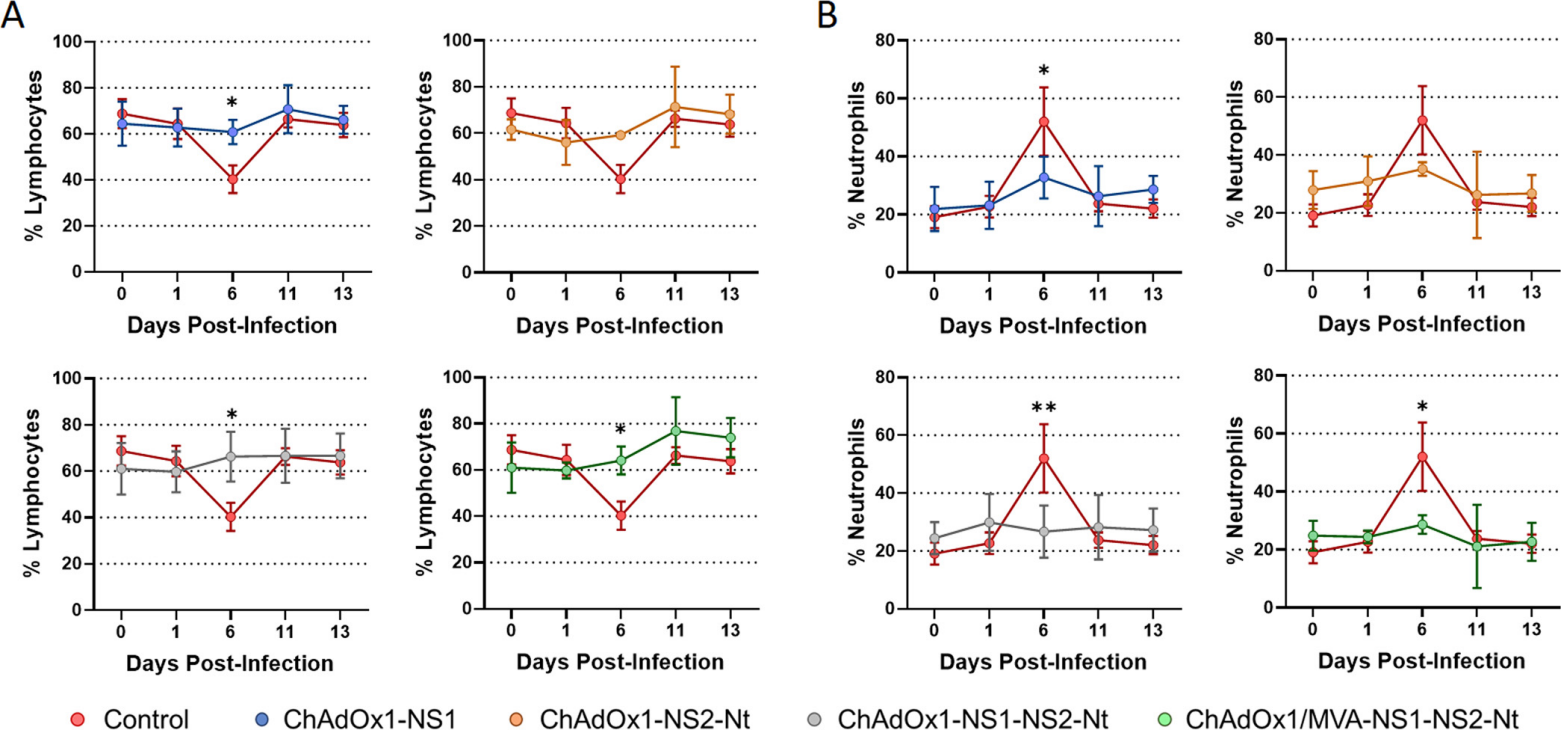
Percentages of lymphocytes and neutrophils in blood from immunized sheep after challenge with BTV-4M. Blood of nonimmunized and immunized sheep were analyzed in an autohematology analyzer (BC-5300 Vet; Mindray, China) and the percentage of lymphocytes (A) and neutrophils (B) based on the total white blood cells were analyzed at days 0, 1, 6, 11, and 13 postinfection. Points indicate the mean value of each group and error bars represent *SD*. * *P value*  <  0.05, ** *P value*  <  0.002, using two-way ANOVA (post hoc Tukey test for multiple comparisons).

BTV infection generates hematological changes in animals including lymphopenia and neutrophilia (41). Nonimmunized sheep suffered lymphocytopenia and neutrophilia at day 6 postinfection followed by the subsequent re-establishment of the normal percentage of lymphocytes (Fig. 9A) and neutrophils (Fig. 9B) in blood thereafter. Sheep immunized with a single dose of ChAdOx1-NS1 or ChAdOx1-NS2-Nt also experimented a depletion of lymphocytes as well as a rise in their levels of neutrophils in blood at 6 d.p.i., but lessened when compared with the control group. In parallel, total percentages of circulating lymphocytes and neutrophils of animals immunized with ChAdOx1-NS1-NS2-Nt and ChAdOx1/MVA-NS1-NS2-Nt remained steady throughout the experiment, which indicates that these vaccine candidates prevented sheep from developing lymphopenia and neutrophilia. All these data indicate that despite the fact that the immunization of sheep with a single dose of ChAdOx1-NS1-NS2-Nt improves the protection conferred by ChAdOx1-NS1 against BTV, the heterologous prime-boost strategy ChAdOx1/MVA-NS1-NS2-Nt confers a better protection against a BTV challenge reducing the level and period of viremia and the presence of macroscopic lesions induced by BTV infection.

### Cell mediated immune responses in sheep.

**FIG 10:**
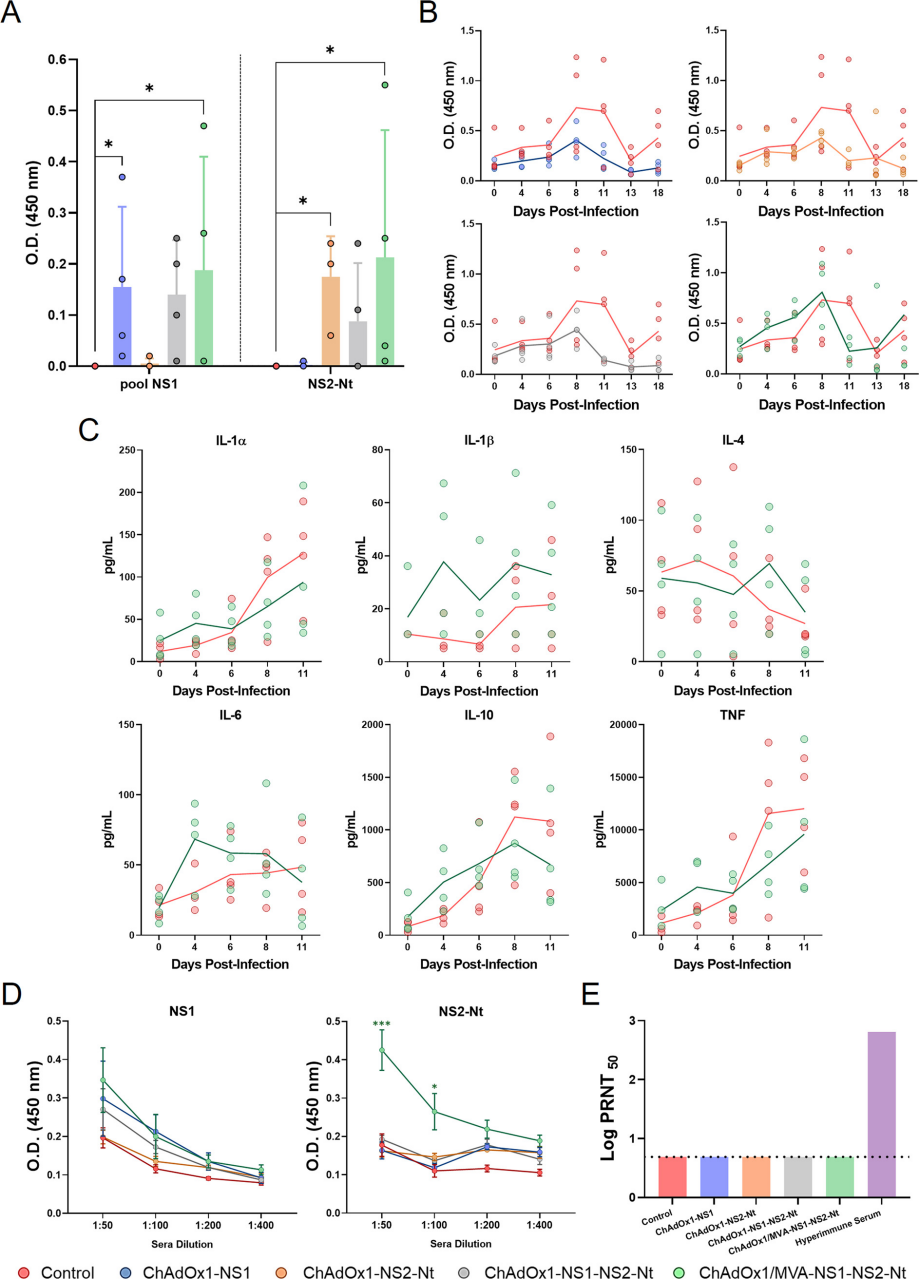
Immunogenicity of vaccine candidates in sheep. (A) IFN-γ levels measured in prechallenge sheep plasma by capture ELISA. Sera from immunized sheep were stimulated for 72 h 4 weeks postprime (prime-only) or 4 weeks postboost (ChAdOx1/MVA-NS1-NS2-Nt) with 10 μg/mL of a pool of NS1 peptides, 10 μg/mL of NS2-Nt or left untreated. Points represent individual values for each sheep, bars represent the mean values of each group and error bars represent *SD*. Asterisks denote significant differences between stimulated samples of immunized and nonimmunized control mice (*P*  <  0.05) (The Mann–Whitney U test). (B) IFN-γ levels measured in postchallenge sheep plasma by capture ELISA. Points represent individual values for each sheep and lines represent the mean values of each group. Data were analyzed by two-way ANOVA (post hoc Tukey test for multiple comparisons). (C) Cytokine presence in plasma from ChAdOx1/MVA-NS1-NS2-Nt immunized sheep and nonimmunized sheep after challenge. Points represent individual values for each sheep and lines represent the mean values of each group. Data were analyzed by two-way ANOVA (post hoc Tukey test for multiple comparisons). (D) Induction of IgG NS1 and NS2-Nt antibodies by indirect ELISA in vaccinated animals. Sera dilutions are indicated in the *x* axis. Points represent the mean values of each group and error bars represent *SD*. Asterisks denote significant differences between immunized and control sheep. * *P value*  <  0.05, *** *P value*  <  0.001, using two-way ANOVA (post hoc Tukey test for multiple comparisons). (E) Neutralizing antibodies titers against BTV-4M in immunized sheep by plaque reduction neutralization assay. Cut-off: 0.69 (log_10_ 5). Hyperimmune serum: serum from sheep infected with BTV-4M. Bars represent the mean values of each group. Data were analyzed by the Mann–Whitney U test.

The induction of cellular immune responses among the different immunized groups was checked at different time points pre- and postchallenge using an IFN-γ capture-ELISA. To determine the capability of these vaccine candidates to induce a cellular immune response in sheep, we stimulated sheep peripheral blood cells 8 weeks postprime (prime-only strategies) or 6 weeks postboost (ChAdOx1/MVA-NS1-NS2-Nt) with a pool of NS1 peptides or with the recombinant protein NS2-Nt for 72 h (Fig. 10A). IFN-γ levels were undetectable in nonimmunized animals after blood stimulation with NS1 peptides or NS2-Nt. In contrast, significant detectable levels of IFN-γ were detected in blood from animals immunized with the prime-only strategies ChAdOx1-NS1 (*P*  =  0.03; mean; OD_450_ = 0.155) and ChAdOx1-NS2-Nt (*P*  =  0.03; mean OD_450_ = 0.175) after stimulation. Interestingly, although IFN-γ was detected in plasma from sheep immunized with ChAdOx1-NS1-NS2-Nt after blood stimulation with NS1 peptides or NS2-Nt, it was nonsignificant compared with the nonimmunized animals. In any case, immunization with ChAdOx1/MVA-NS1-NS2-Nt lead to significant IFN-γ levels in plasma after stimulation of blood with NS1 peptides (*P*  =  0.03; mean OD_450_ = 0.1875) or protein NS2-Nt (*P*  =  0.03; mean OD_450_ = 0.2125), which highlights the capability of the vaccine candidate to prompt a cellular immune response specific of both BTV antigens.

We also evaluated the cellular immune response during infection with BTV (Fig. 10B). Postchallenge plasma from nonimmunized sheep displayed stable IFN-γ levels during the first 6 days postinfection followed by an increased at day 8 postinfection that persisted until day 11 postchallenge. Sheep immunized with a single dose of rChAdOx1 (ChAdOx1-NS1, ChAdOx1-NS2-Nt, or ChAdOx1-NS1-NS2-Nt) did not exhibit such increase in IFN-γ levels in plasma. On the contrary, they were steady throughout the experiment. However, IFN-γ levels did arise more drastically in plasma from sheep subjected to the prime-boost immunization with ChAdOx1/MVA-NS1-NS2-Nt compared with the control group, where elevated although not statistically significant plasmatic levels of IFN-γ were detected as early as 4 days postchallenge with a peak at 8 d.p.i. and a sharp drop in subsequent days. Afterwards, in light of the postchallenge IFN-γ levels exhibited by the prime-boost ChAdOx1/MVA-NS1-NS2-Nt immunization group, we further analyzed plasma samples for the presence of cytokines after challenge (Fig. 10C). At day 4 postinfection, and coinciding with the peak on viremia levels, higher (although not statistically significant) levels of IL-1α, IL-1β, IL-6, IL-10, and TNF were detected in plasma of immunized sheep compared to control sheep, and levels of IL-4 were steady throughout the experiment in the ChAdOx1/MVA-NS1-NS2-Nt immunized sheep (Fig. 10C), suggesting an imbalance toward a Th1 phenotype T cell response influenced by this prime-boost.

To confirm that the protection observed in the immunized sheep against BTV was not due to the induction of neutralizing antibodies, the presence of antibodies specific of NS1 and NS2-Nt in prechallenge immunized sheep sera and their ability to neutralize the virus was analyzed. As expected, we observed that antibodies specific of protein NS1 or NS2-Nt were consistently higher in sheep immunized with the prime-boost ChAdOx1/MVA-NS1-NS2-Nt when compared with control and prime-only groups (Fig. 10D), reflecting the superior immunogenicity of the prime-boost compared with prime-only strategies. Besides, no serum showed neutralizing activity against BTV-4 (Fig. 10E), confirming that the protective capability of the vaccine candidates was not due to the induction of neutralizing antibodies.

## DISCUSSION

The control of bluetongue disease through timely and relevant vaccination is achievable. Although current vaccines are effective, they have significant drawbacks that should be addressed such as their inability to distinguish between vaccinated and infected animals (DIVA strategy), their lack of broad protective immunity against multiple BTV serotypes and the necessity for booster doses.

Recent work in our laboratory focused on the generation of vaccines capable of inducing cross-protective cellular immune responses against multiple BTV serotypes and showed that the BTV antigen NS1 singly expressed by a MVA vector, following a homologous prime-boost immunization, can induce significant protective efficacy against the infection with different BTV serotypes (1, 4, 8, and 16) (22). Furthermore, we showed that a robust T cell mediated immune response was elicited after a single immunization with ChAdOx1 or a heterologous prime-boost combining MVA and ChAdOx1 expressing NS1 or the N-terminus region of NS1 (NS1-Nt) in IFNAR(-/-) mice and sheep, conferring a durable full protection in mice against BTV-8 and BTV-4M and partial protection in sheep against the virulent reassortant BTV-4M (39).

To improve the efficacy of the cellular immune response elicited by NS1 delivered by MVA or ChAdOx1 viral vectors, we included the N-terminal half of protein NS2 (NS2-Nt), highly conserved among BTV serotypes, in the vaccine composition. NS2 has been reported to induce cross-serotype helper T cell and cytotoxic T cell responses in cattle (42, 43). The *in silico* study conducted here showed the presence of theoretical CD8^+^ T-cell epitopes in the N-terminal half of the NS2 protein. In this sense, we confirmed that the proteins NS2 and NS2-Nt but not NS2-Ct delivered by MVA were able to stimulate a specific cellular immune response in IFNAR(-/-) mice. When we analyzed the protection induced by NS2-Nt delivered by MVA or ChAdOx1, we observed that a single dose partially protected against a lethal dose of BTV-4M and full protection was achieved after the prime-boost ChAdOx1/MVA only expressing NS2-Nt, bearing out its potential protective role against BTV. Furthermore, a single dose of MVA-NS1-NS2-Nt or ChAdOx1-NS1-NS2-Nt improved the protection elicited by MVA-NS1 or ChAdOx1-NS1, preventing viremia after infection with BTV-4M. Importantly, the immunization of IFNAR(-/-) mice with the heterologous prime-boost ChAdOx1/MVA-NS1-NS2-Nt conferred protection not only against a homologous lethal challenge with BTV-4M, but also against the heterologous infections with BTV-1 and BTV-8.

Apart from neutralizing antibodies, it is well known the importance of cytotoxic T cells in protection against BTV and they are assumed to be involved in cross-serotype protection (16). The analysis of sera neutralizing activity induced by these two BTV antigens showed that neither NS1 nor NS2-Nt triggered neutralizing antibodies. Instead, animals immunized with ChAdOx1/MVA-NS1, ChAdOx1/MVA-NS2-Nt, or ChAdOx1/MVA-NS1-NS2-Nt developed strong cytotoxic CD8^+^ T-cell responses against NS1, NS2-Nt, or both proteins, respectively. This observation suggests that the protection observed in the immunized mice after challenge relied on the observed CD8^+^ T-cell responses. Although previous studies showed that cytotoxic CD8^+^ T-cell responses raised against NS1 protein play a key role in multiserotype protective immunity against BTV (22, 23, 27, 39, 40), here we demonstrate that protein NS2 also elicits a protective CD8^+^ T-cell response against BTV-4M in IFNAR(-/-) mice after immunization with ChAdOx1/MVA-NS2-Nt or ChAdOx1/MVA-NS1-NS2-Nt strategies. Despite that both BTV antigens were able to induce a CD8^+^ T-cell immune response after immunization, the study of the phenotype of the memory BTV-specific CD8^+^ T cell subpopulations evinced slight differences between these two antigens. Restimulation with the NS1 immunodominant peptide p152 predominantly led to high levels of TEM and TE and very low levels of TCM cells. Conversely, restimulation of splenocytes with NS2-Nt led to a more pronounced TCM phenotype, especially in the case of ChAdOx1/MVA-NS2-Nt immunization group. The aim of T cell vaccines is the generation of durable CD8^+^ T cells with the potential to recognize and resolve infections. TCM and TEM circulate and provide body wide immune surveillance (44). TEM can mount faster cytotoxic responses than TCM, which may relate to superior protection as described in vaccinia virus infection. However, TCMs are finer resolving viral infections that target the lymph nodes and spleen, such as lymphocytic choriomeningitis virus (LCMV), probably due to their higher proliferative potential (45, 46). Furthermore, central memory cells are needed to reestablish the population required for virus clearance (47). Because BTV infects lymphoid tissues, the increase in the TCM subpopulation due to the inclusion of NS2-Nt in the vaccine composition would improve the protection induced by NS1 alone. The kinetics of viral replication or the spread of the infection influence the relative importance of TEM and TCM in mediating protective immunity (44). Although more studies will be necessary to confirm it, the diversification of the antigen-specific memory CD8^+^ T cells subpopulations elicited by NS2-Nt suggests a likely improvement in the long lasting protection previously observed in animals immunized with ChAdOx1/MVA-NS1 (39).

ChAdOx1 is a chimpanzee adenovirus vector platform that has been employed in the development of vaccine candidates for several pathogens. A single dose of ChAdOx1-vectored vaccine fully protects against Rift Valley fever virus, Middle East respiratory syndrome coronavirus, Lassa virus, or Zika virus in animal models of infection (35, 48–51). In the same vein, we also observed the efficacy of a single dose of the recombinant ChAdOx1 as IFNAR(-/-) mice immunized with a single dose of ChAdOx1-NS1-NS2-Nt were fully protected against BTV-4M, showing an aviremic status throughout the experiment. Similarly, sheep immunized with a single dose of ChAdOx1-NS1-NS2-Nt exhibited a shorter viremic status after BTV challenge compared with sheep immunized with ChAdOx1-NS1, ChAdOx1-NS2-Nt, or nonimmunized. Despite the fact that ChAdOx1-NS1-NS2-Nt immunized sheep did display detectable levels of viremia during the first days of infection, postmortem evaluation showed only mild and unspecific lesions whereas nonimmunized control sheep presented specific lesions of BTV infection such as hyperaemia of oral mucosa and gums, petechial hemorrhages on the papillary muscles of the left ventricle, sub-intimal petechial hemorrhages in the pulmonary artery, or mild pulmonary congestion (52–54). Additionally, ChAdOx1-NS1-NS2-Nt also prevented sheep from developing hematological changes characteristic of BTV infection, including lymphopenia and neutrophilia (41).

Although a single immunization with ChAdOx1-NS1-NS2-Nt partially protected sheep against BTV-4M, several studies using the ChAdOx1/MVA prime-boost regimen have shown an increase in the level of protection against a diverse range of pathogens such as hepatitis C virus (HCV) (55), Ebola virus (31), and respiratory syncytial virus (RSV) (56), compared with a single vector prime (57). Besides, it has been reported that homologous prime-boost strategies with ChAdOx1 failed to boost T cell responses in recent SARS-CoV-2 field trials and experimental infections in mice (58–60). Conversely, previous works supported the augmentation of T cell-mediated responses by MVA after a ChAdOx1 prime when combined in heterologous prime-boost strategies in both mice and human (55, 61–63). In the present study, the administration of a booster dose of MVA-NS1-NS2-Nt after a prime of ChAdOx1-NS1-NS2-Nt optimized the protection conferred in sheep by a single dose of ChAdOx1-NS1-NS2-Nt against BTV-4M. Particularly, this booster dose led to lower viremia levels during the first days of BTV infection and was successful to shorten the viremic period after infection when compared with nonimmunized and ChAdOx1-NS1-NS2-Nt immunized sheep. Previous results showed lower viremia levels in sheep immunized with ChAdOx1/MVA-NS1 after challenge with BTV-4M but the period of viremia was similar than the nonimmunized animals (39). The inclusion of NS2-Nt in the vaccine composition improved the protection speeding up the viral clearance. Additionally, hematological changes due to BTV infection were not detected nor macroscopic lesions could be identified in postmortem samples of these immunized animals. Besides, the analysis of the histological lymph node of ChAdOx1/MVA-NS1-NS2-Nt-immunized sheep showed that the LN architecture (data not shown) was preserved after BTV-4M infection, suggesting that this prime-boost immunization strategy not only promotes a faster viral clearance and reduction of the period and level of viremia but also protects from the pathology produced by a BTV infection in the natural host.

Sheep immunization lead to significant IFN-γ levels after stimulation of blood with NS1 peptides or protein NS2-Nt which demonstrates the capability of the vaccine candidate to elicit a cellular immune response specific of both BTV antigens. Analogous to our observation in IFNAR(-/-) mice, the protection observed in sheep immunized with ChAdOx1-NS1-NS2-Nt or ChAdOx1/MVA-NS1-NS2-Nt occurred in the absence of nAbs. Furthermore, after BTV infection, a faster induction of IL-1α, IL-1β, IL-6, IL-10, and TNF was detected in immunized animals compared with the nonimmunized control sheep, suggesting an imbalance toward a Th1 phenotype T cell response influenced by this prime-boost.

The ideal BTV vaccine should be broadly protective or tailor-made to anticipate the circulation of multiple serotypes (15). To achieve broad protection, three pentavalent live attenuated vaccine cocktails are in use in Africa (64). Although this vaccine induces broad protection against BTV, it can cause clinical disease, reversion to virulence, spreading by midges, possible reassortment events between different LAVs or with wild-type BTV, and it is not a DIVA vaccine (15). Recent pentavalent DISA vaccines, based on the in-frame 72-aa deletion in Seg-10 (NS3/NS3a), overcome some of these weaknesses of pentavalent LAVs vaccines. This vaccine is safe, DIVA, and protective against multiple BTV serotypes. Pentavalent DISA12348 vaccine has shown sterile immunity in sheep and cattle for BTV serotypes 2 and 8 by lack of viremia and protection was achieved for serotypes 1 and 4 based on induction of neutralizing antibodies (65). Although it cannot be ruled out that part of the protection generated by these vaccines can be due to an induction of cellular responses, both vaccine approaches base their protection on the induction of high titers of neutralizing antibodies. In contrast, MVA and ChAdOx1-vectored vaccines designed to simultaneously express the immunogenic NS1 protein and/or NS2-Nt base their multiserotype protective capacity on a strong induction of CD8^+^ T cell responses against these two BTV proteins highly conserved among serotypes. The heterologous prime-boost vaccination strategies using two different viral vector vaccines promotes potent T-cell mediated immunity against the antigens of interest (66). In this way, the heterologous prime-boost strategy combining both MVA and ChAdOx1 expressing NS1 and/or NS2-Nt increases the cellular immune response against NS1 and NS2 specifically, something that does not occur in the context of the natural BTV infection.

There are many examples in experimental animal models where vaccine-induced CD8^+^ T cells protect against intracellular pathogens, like LCMV and influenza virus (67–70). As it has been previously stated, the vaccine strategies ChAdOx1-NS1-NS2-Nt and ChAdOx1/MVA-NS1-NS2-Nt elicited protection in immunized sheep although they did not confer sterilizing immunity, which is not abnormal as long as CD8^+^ T cells do not prevent completely the infection because usually they recognize and respond to infected cells, but they can eventually outcome rapid virus clearance (71). We observed a reduction in the levels of viremia and a shortening of the viremic period motivated by ChAdOx1-NS1-NS2-Nt and ChAdOx1/MVA-NS1-NS2-Nt immunization strategies. It is important to note that viremia was measured from systemic blood samples after bleeding of sheep in the jugular vein. As pointed out by Veronesi et al., the viral load found in systemic blood could be dissimilar to that found on skin periphery (72), probably lower if we attend to the work conducted by Melzi et al., in which the concentration of viral RNA in systemic blood was between 100 and 1,000 times higher than that found in skin from day 5 postinfection (73). Experimental infections have shown that Culicoides infection rates are very low, even when blood-fed on experimentally infected sheep at peak viremia levels (72). If we consider that the efficiency of Culicoides infection is dose-dependent and the 50% midge alimentary infective dose (MAID50) has been estimated to a blood meal titer between 10^5^ and 10^6^ TCID50/mL (74–76), immunization with ChAdOx1-NS1-NS2-Nt or ChAdOx1/MVA-NS1-NS2-Nt probably can contribute to minimize Culicoides infection rates, thus impairing virus transmission.

In summary, this study demonstrates that NS2-Nt is an applicable antigen in vaccines against BTV and its combination with NS1 in a single dose with ChAdOx1 or a heterologous prime-boost ChAdOx1/MVA induces a potent BTV CD8^+^ T cell immune response that promotes a faster viral clearance and protects sheep against BTV infection. Moreover, this safe and adjuvant-free vaccine candidate is compatible with a DIVA strategy and, more importantly, is able to confer protection against multiple BTV serotypes, one of the major challenges in vaccination against BTV.

## MATERIALS AND METHODS

### Ethics statement.

Animal experimental protocols were approved by the Ethical Review Committee at the INIA-CISA and Comunidad de Madrid (Permit number: PROEX 172/17) in strict accordance with EU guidelines 2010/63/UE about protection of animals used for experimentation, and other scientific purposes and Spanish Animal Welfare Act 32/2007.

### Cell lines and viruses.

Chicken embryo fibroblasts (DF-1) (ATCC, Cat. No. CRL-12203) and human embryo kidney cells (HEK293) (ATCC, Cat. No. CRL-11268G-1) were grown in Dulbecco’s Modified Eagle’s medium (DMEM) (Biowest, Nuaillé, France) supplemented with 2 mM glutamine (Gibco, Waltham, MA, USA) and 10% fetal calf serum (FCS) (Gibco, Waltham, MA, USA). Green monkey kidney cells (Vero) (ATCC, Cat. No. CCL-81) were grown in DMEM supplemented with 2 mM glutamine and 5% FCS.

BTV serotype 1 (ALG2006/01) (BTV-1), BTV serotype 4 Morocco strain (MOR2009/09) (BTV-4M), and BTV serotype 8 BEL/2006) (BTV-8) were used in the experiments. BTV-4M strain is a reassortant strain between BTV-1 (segments 1, 4, 5, 7, 9, 10) and BTV-4 (segments 2, 3, 6, 8) isolated from sheep blood in Kenyon (KC) insect cells (77, 78). Virus stocks and titrations were performed by standard methods previously described (79).

### *In silico* NS2 T-CD8 epitope prediction.

Amino acid sequence for the nonstructural NS2 protein (NCBI accession number: KP821792) was analyzed using two prediction algorithms available on the web: Immune Epitope DataBase (IEDB Analysis Resource) (www.iedb.org) and SYFPEITHI (http://www.syfpeithi.de/) for the H-2-Db MHC class I for 129/Sv mice to identify theoretical T-CD8 epitopes that could be good binders to H-2-Db MHC. Theoretical T-cell epitopes were chosen by a combination of the best score in these databases.

### Generation of recombinant MVA vaccine vectors.

The MVA transfer plasmids pSC11 containing NS2, NS2-Nt, or NS2-Ct were generated and used to produce MVA-NS2, MVA-NS2-Nt, or MVA-NS2-Ct. Briefly, to generate plasmid pSC11 containing NS2, NS2-Ct, or NS2-Nt, these genes were amplified from total RNA extracted from BTV-4 (SPA2004/02) infected Vero cells with primers specified in Table 2, as previously described (80). The restriction site XmaI was introduced at 5′ and 3′ ends of each PCR product. Subsequently, the DNA inserts were digested with XmaI and cloned into the MVA transfer plasmid pSC11 previously digested with the same restriction enzyme and dephosphorylated. Plasmids pSC11-NS2, pSC11-NS2-Nt, and pSC11-NS2-Ct were transfected in DF-1 cells infected with MVA-wt at a multiplicity of infection (MOI) of 1 using Lipofectamine™ 3000 transfection reagent (Invitrogen™, CA, USA), following the protocol facilitated by the manufacturer. This allows recombination of the transgene and the marker *LacZ* with the MVA genome in the native TK ORF. Cell cultures were harvested at 48 h.p.i. and selection of rMVAs was performed by plaque assay in the presence of X-Gal. Complementary, rMVAs were cloned at least four times by plaque assay for a greater purification.

**TABLE 2:** Primers designed to generate rMVA, rChAdOx1, and rBac

Primer	Sequence
Fw-XmaI-NS2-1^*a*^	5′ CGcccgggATGGAGCAAAAGCAACGTAG 3′
Rs-XmaI-NS2-1062*	5′ CGcccgggCTAAACGCCGACCGGCAATA 3′
Rs-XmaI-NS2-531*	5′ CGcccgggCTACGCCACGCTTTGAACTTG 3′
Fw-XmaI-NS2-532*	5′ CGcccgggATGCCAAGGGAAGAATCACGC 3′
Fw-2A-NS1^*b*^	5′-cttgctgcgcgtatgtctcagatgtggatggaatatGCCCCTGTGAAGCAG-3′
Rs-2A-NS2-Nt^*b*^	5′-gttgcttttgctccatGGGGCCAGGGTTGCTTTCCACGTCGCCGGC-3′
Fw-2A-NS2-Nt^*b*^	5′-GCCGGCGACGTGGAAAGCAACCCTGGCCCCatggagcaaaagcaac-3′
Rs-NS2-Nt-p1990^*b*^	5′-GGGCCCTCTAGATGCATGCTCGAGCGGCCGCctacgccacgctttg-3′
Fw-BamHI-NS2 1^*c*^	5′-CgggatcCGATGGAGCAAAAGCAACGTAG-3′
Rs-NotI-NS2-531^*c*^	5′-CGgcggccgcCTACGCCACGCTTTGAACTTG-3′
Fw-BamHI-NS2-532^*c*^	5′-CgggatcCGATGCCAAGGGAAGAATCACGC-3′
Rs-NotI-NS2-1062*^c^*	5′-CGgcggccgcCTAAACGCCGACCGGCAATA-3′

a Primers designed to generate pSC11-NS2, pSC11-NS2-Nt, and pSC11-NS2-Ct. XmaI restriction site is represented by lowercase letters.

b Primers designed to generate rChAdOx1. Complementary sequences are shown in capital letters. Lowercase letters represent the overlapping sequences.

c Primers designed to generate BAC-NS2-Nt and BAC-NS2-Ct. BamHI and NotI restriction sites are represented by lowercase letters.

Thereafter, we generated rMVAs containing NS1 or NS2-Nt placed in the F13L locus, and a rMVA simultaneously expressing NS1 and NS2-Nt. For this purpose, plasmids pMVA containing NS1 or NS2-Nt genes from BTV-4 (SPA2004/02) were constructed. Shortly, previously generated plasmid pcDNA3-NS1 (22, 80, 81) was digested with XhoI and BamHI restriction enzymes and the DNA insert was cloned into the MVA transfer plasmid pMVA-β-Gus (82) previously digested with the same restriction enzymes. In parallel, NS2-Nt was amplified from plasmid pSC11-NS2-Nt with primers Fw-5′-CGGctagCGAGCAAAAGCAACGTAG-3′ and Rs-5′-CCGgatcCCTACGCCACGCTTTGAACTTGAAGCCG-3′. The restriction sites NheI and BamHI were introduced at the 5′ and 3′ ends of the PCR product. The DNA insert was digested with NheI and BamHI and introduced in the MVA transfer plasmid pMVA-β-Gus previously digested with the same restriction enzymes. Subsequently, plasmids pMVA-NS1 or pMVA-NS2-Nt were transfected in DF-1 cells infected with MVAΔF13L that encodes *dsRed* marker instead of the native F13L ORF at an MOI of 1. Cell cultures were harvested at 48 h.p.i. and rMVAs were purified by plaque-picking and fluorescent selection in a Zeiss Axio fluorescence microscope (Zeiss, Oberkochen, Germany). Complementary, rMVAs were cloned at least four times by plaque assay for a greater purification.

Consecutively, MVA transfer plasmid pSC11 containing NS2-Nt was used to generate MVA-NS1-NS2-Nt. Briefly, previously generated plasmid pSC11 containing NS2-Nt was transfected in DF-1 cells infected with the MVA-NS1 generated in the prior step at an MOI of 1. Cell cultures were harvested at 48 h.p.i. and selection of rMVAs was performed by plaque assay in the presence of X-Gal. Dual MVAs were cloned four times by plaque isolation assay.

### Generation of recombinant adenoviral vaccine vectors.

In order to generate rChAdOx1 expressing NS1, NS2-Nt, and NS1-NS2-Nt, a 1990_pENTR4-LPTOS (p1990) adenovirus entry vector containing NS1 or NS2-Nt genes from BTV-4 (SPA2004/02) were generated. Briefly, previously generated plasmids pcDNA3-NS1 (22, 80, 81) and HTA-NS2-Nt were digested with EcoRI and NotI, or with BamHI and NotI, respectively, and the DNA inserts were cloned into the plasmid p1990 previously digested with the same restriction enzymes. To generate p1990 adenovirus entry vector containing NS1 from BTV-4, along with 2A picornavirus gene and NS2-Nt gene from BTV-4, previously cloned plasmid p1990-NS1 was used. 2A gen from plasmid pcDNA3-2A (facilitated by The Jenner Institute Viral Vector Core Facility of the University of Oxford) was amplified using Fw-2A-NS1 that miss stop codon out of NS1 and Rs-2A-NS2-Nt primers (Table 2). NS2-Nt gen from plasmid p1990-NS2-Nt was amplified using Fw-2A-NS2-Nt and Rs-NS2-Nt-p1990 primers (Table 2). Plasmid p1990-NS1 was digested with NotI and dephosphorylated. In order to generate a single shuttle vector containing NS1-2A-NS2-Nt genes, ligation of pENTR-LP-NS1, 2A, and NS2-Nt amplification products was conducted by using the NEBuilder HiFi DNA Assembly mix (New England Biolabs, Ipswich, MA, USA), following manufacturer’s protocol. Plasmids p1990-NS1, p1990-NS2-Nt, and p1990-NS1-2A-NS2-Nt were sequenced and sent to The Jenner Institute Viral Vector Core Facility at the University of Oxford for rChAdOx1 generation and purification.

### Indirect immunofluorescence microscopy.

DF-1 cells were grown in glass coverslips and infected with MVA-NS1, MVA-NS2, MVA-NS2-Nt, MVA-NS2-Ct, or MVA-NS1-NS2-Nt at an MOI of 1, respectively. HEK293 cells were grown in a 96-well plaque and infected with ChAdOx1-NS1, ChAdOx1-NS2-Nt, or ChAdOx1-NS1-NS2-Nt at an MOI of 0.5, respectively. Twenty-four hours (rMVA) or 18 h (rChAdOx1) after infection, cell monolayers were fixed for 15 min with 40% acetone-methanol. Fixed cells were blocked with 20% phosphate-buffered saline–fetal bovine serum (20% FBS-PBS) (20% blocking solution) for 60 min at room temperature (RT). DF-1 cells were then incubated overnight at 4°C with a mouse hyperimmune serum against ChAdOx1-NS1 (obtained from an *in vivo* infection [39]) (1:500) or the NS2 BTV specific monoclonal antibody (MAb) 23H6 (Eurofins INGENASA, Madrid, Spain) (1:500), diluted in PBS-FBS 20%. HEK293 cells were incubated overnight at 4°C with a sheep hyperimmune serum against MVA-NS1 (obtained from an *in vivo* infection) (1:500) and the NS2 BTV MAb 23H6 (Eurofins INGENASA, Madrid, Spain) (1:500) diluted in PBS-FBS 20%. After three serial washing steps with PBS, DF-1 cells were incubated for 30 min at RT with Alexa Fluor 488 goat conjugated anti-mouse IgG (Invitrogen™, German Town, MD, USA) (1:1,000) or 594 goat conjugated anti-mouse IgG (Invitrogen™, German Town, MD, USA) (1:500). For HEK293 cells, Alexa Fluor 488 goat conjugated anti-mouse IgG (Invitrogen™, German Town, MD, USA) (1:1,000) and 594 goat conjugated anti-sheep IgG (Invitrogen™, German Town, MD, USA) (1:500) were incubated for 30 min at RT after three serial washing steps. Coverslips with infected DF-1 cells and wells with infected HEK293 cells were washed three times with PBS and once with PBS-DAPI (1:10,000) and visualized in a Zeiss Axio fluorescence microscope (Zeiss, Oberkochen, Germany). Adobe Photoshop CS5 Extended (Adobe Systems, CA, USA) was used afterwards for image editing.

### Generation of recombinant baculovirus expressing NS2-Nt or NS2-Ct.

To obtain recombinant proteins NS2-Nt and NS2-Ct, recombinant baculoviruses (rBac) Bac-NS2-Nt and Bac-NS2-Ct were generated using the Bac-to-Bac system (Invitrogen, Barcelona, Spain), following the supplier protocols. Briefly, plasmid pSC11-NS2 containing the sequence coding for NS2 protein of BTV serotype 4 was used to amplify the sequence of NS2-Nt and NS2-Ct with specific primers (Table 2). The PCR products were then cloned into plasmid pFastBac1 (Invitrogen, Barcelona, Spain) digested with BamHI and NotI to obtain the transfer vectors HTA-NS2-Nt and HTA-NS2-Ct that were used to generate the recombinant baculoviruses that express the NS2-Nt or NS2-Ct BTV proteins in insect high five cells (H5), named Bac-NS2-Nt and Bac-NS2-Ct. These proteins were purified using the ProBond Purification System (Invitrogen, Barcelona, Spain) following the procedure indicated by the manufacturer for protein purification under native (NS2-Ct) or hybrid native-denaturing (NS2-Nt) conditions.

### Western blot analysis.

DF-1 and HEK293 cells were infected with the previously generated rMVAs (MOI  =  0.1) or rChAdOx1 (MOI  =  0.1), respectively, or were mock infected. At 24 h.p.i., cells were harvested, washed in PBS containing protease inhibitor cocktail (Sigma-Aldrich, St. Louis, MO, USA), and lysed with RIPA Buffer (Santa Cruz Biotechnology, Dallas, TX, USA). Extracts were then sonicated for 2 min and proteins were resolved in 12% SDS-PAGE and blotted onto a nitrocellulose membrane. After a blocking step with 5% low fat dry milk in TBS Tween 20 (TBST) (blocking buffer), mouse hyperimmune serum against BTV (1:500) or α-BTV NS2 MAb 23H6 (1:500) were applied to membranes in TBST-Milk 5% and incubated overnight at 4°C. Bound antibody was detected with horseradish peroxidase-conjugated rabbit anti-mouse antibody (Sigma-Aldrich, San Louis, MO, USA) diluted in TBST-Milk 5% (1:10,000) and the ECL detection system (Amersham^TM^ Pharmacia Biotech, Buckinghamshire, UK).

### Mice and sheep.

Type I interferon receptor defective mice (IFNAR (-/-)) on a 129 Sv/Ev background and sheep (Spanish “Churra” sheep breed) were used for the studies. All mice and sheep used were matched for age (8 weeks, and 6 months, respectively). Mice and sheep were housed under pathogen-free conditions and allowed to acclimatize to the biosafety level 3 (BSL3) animal facilities at the Animal Health Research Center (INIA-CISA), Madrid, before use.

### *Ex vivo* IFN-γ ELISPOT.

Groups of IFNAR(-/-) mice (*n*  =  4) were immunized following a homologous prime-boost regimen with MVA-NS2, MVA-NS2-Nt, or MVA-NS2-Ct (10^7^ PFU per mouse), 2 weeks apart, or nonimmunized. All animals were sacrificed at 15 days postboost, and their spleens were harvested for analysis by ELISPOT.

ELISPOT plates containing Immobilon-P membranes (Millipore) were first activated with 70% ethanol for 30 s, coated with 5 μg/mL of anti-mouse IFN-γ MAb AN18 (BD Biosciences, San Jose, CA, USA) in 50 μL of carbonate–bicarbonate buffer, using 100 μL/well and stored overnight at 4°C. Subsequently, plates were blocked for 1 h at 37°C with cell culture medium. A total of 5 × 10^5^ splenocytes were added to the well and stimulated with 10 μg/mL of purified NS2-Nt or with 10 μg/mL of purified NS2-Ct. Plates were incubated at 37°C and 5% CO_2_ for 18 h to 20 h. As a positive control, phytohemagglutinin (PHA) at 5 μg/mL was used. Cells were washed six times and incubated for 2 h at RT with 50 μL/well of biotinylated rat anti-mouse IFN-γ R46A2 (BD Biosciences, San Jose, CA, USA) diluted to 1 μg/mL in PBS. This was followed by incubation with HRP streptavidin (BD Biosciences, San Jose, CA, USA) diluted to 1 μg/mL in PBS for 1 h at RT. Subsequently, a further incubation for 15 min with 3-amino-9-ethylcarbazole (AEC) (BD Biosciences, San Jose, CA, USA) was performed at RT in dark conditions. The plates were then washed intensively with tap water and dried at RT overnight. Once the membranes were dried, the number of spots in each well was determined using an AID iSpot Reader System (Autoimmun Diagnostika, Strassberg, Germany).

### Mice immunization and challenge.

Three different immunization strategies were evaluated in mice. First, a set of three groups of mice (*n*  =  5) were immunized with a single dose of 2 × 10^7^ PFU per mouse of the rMVAs (MVA-NS1, MVA-NS2-Nt, or MVA-NS1-NS2-Nt). A second set of three groups of mice (*n*  =  5) were immunized with a single dose of 10^8^ IU per mouse of the rChAdOx1 (ChAdOx1-NS1, ChAdOx1-NS2-Nt, or ChAdOx1-NS1-NS2-Nt). A third set of three groups of mice (*n*  =  5) were immunized following a heterologous prime-boost regime consisting of an initial dose of 10^8^ IU per mouse of the ChAdOx1-NS1, ChAdOx1-NS2-Nt, or ChAdOx1-NS1-NS2-Nt (prime) followed by a second dose of 2 × 10^7^ PFU per mouse of MVA-NS1, MVA-NS2-Nt, or MVA-NS1-NS2-Nt (boost), respectively, administered 4 weeks apart. An additional set of two groups of mice (*n*  =  5) were immunized following the heterologous prime-boost ChAdOx1/MVA-NS1-NS2-Nt to conduct the multiserotype experiment. rMVA vectors were inoculated intraperitoneally whereas immunization with the corresponding rChAdOx1 was conducted via intramuscular injection on the right leg of each mouse. For each study, one group of mice (*n*  =  5) was left untreated (control).

Animals were subcutaneously challenged with a lethal dose (10 PFU) of BTV-4M 2 weeks after immunization in the case of those animals given a single dose of rMVA and 4 weeks after immunization in the case of mice given a single dose of rChAdOx1. Animals subjected to the heterologous prime-boost ChAdOx1/MVA-NS1-NS2-Nt strategy were challenged with a lethal dose of BTV-4M (10 PFU), BTV-1 (100 PFU), or BTV-8 (100 PFU), 2 weeks postboost with rMVAs. In all cases, submandibular blood collection was carried out in mice after virus challenge at 3, 5, 7, 10, and 15 d.p.i. for the analysis of viremia.

### Sheep immunization and challenge.

A total of 20 naive healthy sheep (Spanish “Churra” sheep breed), aged 6 months, were acclimated for 7 days at the BSL3 animal facility of the Animal Health Research Center (INIA-CISA) before starting the experiment. All sheep involved in the experiment were negative to BTV by ELISA. Briefly, a set of three groups of sheep (*n*  =  4) was subcutaneously immunized with a single dose of 10^9^ IU of rChAdOx1 (ChAdOx1-NS1, ChAdOx1-NS2-Nt, or ChAdOx1-NS1-NS2-Nt). An additional group of four sheep was immunized following a heterologous primer-boost strategy consisting of an initial dose of 10^9^ of ChAdOx1-NS1-NS2-Nt (prime) followed by a second subcutaneous dose of 10^8^ PFU of MVA-NS1-NS2-Nt (boost) 4 weeks later. A group of sheep was left untreated (control).

Prechallenge blood samples were collected from all animals. Nonimmunized and immunized sheep were subcutaneously challenged with a dose of 10^5^ PFU of BTV-4M 8 weeks after immunization (prime-only) or 6 weeks postbooster (prime-boost). After virus challenge, blood collection for virological analyses was conducted by specialized veterinary personal at 0, 1, 4, 6, 8, 11, 13, and 18 d.p.i. At day 18 postinfection all sheep were euthanized.

### Viremia analysis by RT-qPCR.

Blood samples were collected at 3, 5, 7, 10, and 15 d.p.i. from the submandibular plexus of mice and at 0, 1, 4, 6, 8, 11, 13, and 18 d.p.i. from sheep with EDTA as anti-coagulant. RNA was extracted from 50 μL of blood using TRIzol Reagent (Sigma-Aldrich, St. Louis, MO, USA) following the protocol established by the manufacturer. Viremia was analyzed in duplicate by real-time RT-qPCR specific for BTV segment 5 (encoding for NS1). The real-time RT-qPCR specific for BTV segment 5 was performed using primers and probe described by Toussaint et al. (83). Only Ct values lower than 38 were considered indicative of viremia (positive), according to the cut-off established by Toussaint et al. (83). Sheep and mice blood containing different concentrations of virus were titrated and used as internal standards of the experiment (22).

### Blood measurements.

A multiparameter autohematology analyzer (BC-5300 Vet; Mindray, China) was used to determine the total and differential cell counts in sheep blood for each group and collected into EDTA tubes.

### Evaluation of clinical signs and macroscopic lesions.

After challenge, rectal temperatures and clinical signs were monitored daily. The clinical score used was based on previous protocols (84).

### Macroscopic and histopathological evaluation.

Sheep 12 and 74 (nonimmunized control group), 10 and 16 (ChAdOx1-NS1-NS2-Nt), and 9 and 13 (ChAdOx1/MVA-NS1-NS2-Nt) were euthanized at day 18 postchallenge. Macroscopic lesions were evaluated during necropsies of the bodies using scores based on previous protocols (52).

### *Ex vivo* flow cytometric analysis.

In order to evaluate the cellular immune response in mice, a set of three groups of IFNAR(-/-) mice (*n*  =  4) was subjected to a heterologous prime-boost regimen (ChAdOx1/MVA-NS1, ChAdOx1/MVA-NS2-Nt, and ChAdOx1/MVA-NS1-NS2-Nt). rMVA vectors were inoculated intraperitoneally 4 weeks after immunization with the corresponding rChAdOx1, which was conducted via intramuscular injection on the right leg of each mouse. For this study, one group of mice (*n*  =  4) was left untreated (control). All animals were euthanized at 15 days postboost, and their spleens were harvested for analysis by ICS.

A total of 10^6^ splenocytes per well were stimulated with 5 μg/mL of NS1-152 peptide (9-mer peptide GQIVNPTFI), 5 μg/mL of the NS2-Nt protein, concanavalin A (ConA) as a nonspecific stimulus (4 μg/mL) for 5 h (18 h in the case of NS2-Nt protein), or left untreated in RPMI 1640 medium supplemented with 10% FCS. Six hours before the assay, CD107a/LAMP-1-FITC antibody at 1:10 dilution (Miltenyi, Biotec, Bergisch Gladbach, Germany) and brefeldin A (5 μg/mL) were added. After stimulation, cells were washed with PBS-1%-FBS, stained for the surface markers, fixed with PBS-1%-FBS-1%-Saponine-4%-PFA, permeabilized with PBS-1%-FBS-1%-Saponine, and stained intracellularly using the fluorochrome conjugated antibody IFN-γ–PE (Miltenyi Biotec, Bergisch Gladbach, Germany). Fluorochrome conjugated antibodies CD8-PerCP-Vio700, CD62L-APC, and CD127-FITC (Miltenyi Biotec, Bergisch Gladbach, Germany) were used for the analysis of extracellular receptor molecules. Data were acquired by FACS analysis on a FACSCalibur platform (Becton Dickinson, Franklin Lakes, NJ, USA). Analyses of the data were performed using FlowJo software version x0.7 (Tree Star, Ashland, OR, USA). The number of lymphocyte-gated events was 5 × 10^5^. Lymphocytes were initially gated on the basis of their forward and side scatter properties. Then, CD8^+^ lymphocytes expressing IFN-γ or CD107a were selected for the analysis. Gating strategies used to identify CD8^+^ T-cell populations are showed in the Fig. S1.

### Detection of IFN-γ in plasma samples by capture ELISA.

Immunized and nonimmunized sheep were bled 8 weeks after the prime dose of rChAdOx1 or 6 weeks after the boost with rMVA. Blood from immunized and nonimmunized sheep were stimulated for 72 h with 10 μg/mL of a pool of NS1 peptides (9-mer peptides GQIVNPTFI, SALVNSERV, and IQLINFLRM), with 10 μg/mL of recombinant NS2-Nt, or left untreated in RPMI 1640 supplemented with 10% FCS. Consecutively, blood plasma was extracted. Complementary, sheep were bled at day 0, 4, 6, 8, 11, 13, and 18 postchallenge and blood plasma was extracted. Supernatant IFN-γ levels in pre- and postchallenge plasma samples were analyzed by ELISA. Also, 96-well Costar plates (Sigma-Aldrich, St. Louis, MO, USA) were coated with 2 μg/mL of MAb anti-bovine interferon-gamma (clone MT17.1, Mabtech) and incubated overnight at 4°C. Next day, unspecific binding sites were blocked with PBS-0.05% Tween 20-0.1%-BSA for 1 h at RT. After five washes in PBS-0.05% Tween 20, 50 μL of plasma from each time point sample were incubated for 1 h at 37°C. After washing steps, biotinylated anti bovine IFN-γ (clone MT307, Mabtech) was added at 0.25 μg/mL. Plates were then washed and streptavidin-HRPO (Becton Dickinson, Franklin Lakes, NJ, USA) was used at a 1:1,000 dilution for 1 h at RT. For detection of immunocomplexes, substrate solution 3,3′, 5,5′–tetramethylbencidine liquidsupersensitive (TMB) was added to plates (Sigma-Aldrich, St. Louis, MO, USA). Absorbance values were determined at 450 nm by an automated reader (BMG, Labtech, Victoria, Australia).

### Cytokine analysis.

Nonimmunized and ChAdOx1/MVA-NS1-NS2-Nt blood plasma samples from day 0, 4, 6, 8, and 11 postchallenge were used to analyze supernatant cytokine levels using a multiplex fluorescent bead immunoassay for quantitative detection of sheep cytokines (Millipore’s MILLIPLEX Sheep Cytokine kit, Burlington, MA, USA). Samples were analyzed with a MAGPIX system (Luminex Corporation, Austin, TX, USA). Values of nonstimulated samples were subtracted from values of stimulated samples.

### Detection of antibodies specific of NS1 and NS2-Nt by ELISA.

MaxiSorp plates (Nunc) (Thermo Fisher Scientific, NY, USA) were coated with NS1 (250 ng per well) or NS2-Nt (200 ng per well) purified baculovirus expressed proteins in PBS and incubated overnight at 4°C. Plates were saturated with blocking buffer (PBS-0.05%-Tween 20-5% skim milk). Individual mice or sheep sera diluted in blocking buffer were added and incubated for 2 h at 37°C. After three washes in PBS-0.05% Tween 20, plates were incubated for 1 h at 37°C with an anti-mouse-HRP secondary antibody (Bio-Rad, Hercules, CA, USA) (1:2,000) or with an anti-sheep-HRP secondary antibody (Sigma-Aldrich, San Louis, MO, USA) (1:7,500) in blocking buffer. Finally, after three washes in PBS-0.05% Tween 20, the reaction was developed with 50 μL of TMB (Thermo Fisher Scientific, MD, USA) and stopped by adding 50 μL of 3 N H_2_SO_4_ (Merck, Darmstadt, Germany). Results were expressed as optical densities (ODs) measured at 450 nm.

### Plaque reduction neutralization test.

Two-fold dilutions (from 1:5) of heat inactivated mice or sheep sera (56°C for 30 min) were incubated with 100 PFU of BTV-4 for 1 h at 37°C. Then, samples were inoculated into 12-well plates containing semi-confluent monolayers of Vero cells. Following incubation for 1 h, an agar overlay (DMEM-10%-FBS-0.4%-Noble Agar, Becton Dickinson, MD, USA) was added and plates were incubated for 5 days at 37°C in 5% CO_2_. Plaques were fixed with 10% formaldehyde and visualized with 2% crystal violet-PBS. A 50% plaque reduction neutralization test (PRNT_50_) titer was calculated as the reciprocal (log_10_) of the highest dilution of serum that neutralized 50% of the control virus input. The cutoff is 0.69, log of the reciprocal of the first dilution 1:5.

### Statistical analysis.

Data were analyzed using GraphPad Prism version 8.0.1 (GraphPad Software, San Diego, CA, USA). Survival curves for each immunized group were compared with those of nonimmunized mice in search of statistical differences using log-rank test. Comparisons of mean responses between groups in the viremia analyses were performed using an independent-samples Student's *t* test. Data from the ELISPOT, ICCS, NS1, and NS2-Nt ELISA of mice sera, and PRNT_50_ assays were analyzed using Mann-Whitney non-parametric test. Comparisons of mean responses between groups in the hematology, IFN-γ ELISA, Multiplex cytokine assay, and NS1 and NS2-Nt ELISA of sheep sera assays were conducted by two-way ANOVA with a post hoc Tukey test for multiple comparisons. A *P value* lower than 0.05 was considered significant in all cases.

### Data availability.

All data generated or analyzed during this study are included in the main text and supplementary information. All relevant data are also available upon request from the corresponding authors.

## References

[1] Du TR. 1944. The transmission of blue-tongue and horse-sickness by Culicoides. J Vet Sc Anim Ind 19:7–16.

[2] Maclachlan NJ, Drew CP, Darpel KE, Worwa G. 2009. The pathology and pathogenesis of bluetongue. J Comp Pathol 141:1–16. 10.1016/j.jcpa.2009.04.003. 19476953

[3] Roy P. 2017. Bluetongue virus structure and assembly. Curr Opin Virol 24:115–123. 10.1016/j.coviro.2017.05.003. 28609677

[4] Verwoerd DW, Louw H, Oellermann RA. 1970. Characterization of bluetongue virus ribonucleic acid. J Virol 5:1–7. 10.1128/JVI.5.1.1-7.1970. 4315158 PMC375961

[5] Mertens PP, Brown F, Sangar DV. 1984. Assignment of the genome segments of bluetongue virus type 1 to the proteins which they encode. Virology 135:207–217. 10.1016/0042-6822(84)90131-4. 6328750

[6] Huismans H, van der Walt NT, Cloete M, Erasmus BJ. 1987. Isolation of a capsid protein of bluetongue virus that induces a protective immune response in sheep. Virology 157:172–179. 10.1016/0042-6822(87)90326-6. 3029956

[7] Schulz C, Bréard E, Sailleau C, Jenckel M, Viarouge C, Vitour D, Palmarini M, Gallois M, Höper D, Hoffmann B, Beer M, Zientara S. 2016. Bluetongue virus serotype 27: detection and characterization of two novel variants in Corsica, France. J Gen Virol 97:2073–2083. 10.1099/jgv.0.000557. 27435041

[8] Bumbarov V, Golender N, Jenckel M, Wernike K, Beer M, Khinich E, Zalesky O, Erster O. 2020. Characterization of bluetongue virus serotype 28. Transbound Emerg Dis 67:171–182. 10.1111/tbed.13338. 31469936

[9] Yang H, Gu W, Li Z, Zhang L, Liao D, Song J, Baoxin S, Hasimu J, Li Z, Yang Z, Zhong Q, Li H. 2021. Novel putative bluetongue virus serotype 29 isolated from inapparently infected goat in Xinjiang of China. Transbound Emerg Dis 68:2543–2555. 10.1111/tbed.13927. 33190404

[10] Rushton J, Lyons N. 2015. Economic impact of bluetongue: a review of the effects on production. Vet Ital 51:401–406. 10.12834/VetIt.646.3183.1. 26741252

[11] Alonso C, Utrilla-Trigo S, Calvo-Pinilla E, Jiménez-Cabello L, Ortego J, Nogales A. 2020. Inhibition of orbivirus replication by aurintricarboxylic acid. Int J Mol Sci 21. 10.3390/ijms21197294. PMC7582255 33023235

[12] Mohd Jaafar F, Monsion B, Belhouchet M, Mertens PPC, Attoui H. 2021. Inhibition of orbivirus replication by fluvastatin and identification of the key elements of the mevalonate pathway involved. Viruses 13:1437. 13 10.3390/v13081437. 34452303 PMC8402872

[13] Jiménez-Cabello L, Utrilla-Trigo S, Calvo-Pinilla E, Moreno S, Nogales A, Ortego J, Marín-López A. 2020. Viral vector vaccines against bluetongue virus. Microorganisms 9:42. 10.3390/microorganisms9010042. PMC7823852 33375723

[14] Calvo-Pinilla E, Castillo-Olivares J, Jabbar T, Ortego J, de la Poza F, Marín-López A. 2014. Recombinant vaccines against bluetongue virus. Virus Res 182:78–86. 10.1016/j.virusres.2013.11.013. 24287057

[15] van Rijn PA. 2019. Prospects of next-generation vaccines for bluetongue. Front Vet Sci 6:407. 10.3389/fvets.2019.00407. 31824966 PMC6881303

[16] Andrew M, Whiteley P, Janardhana V, Lobato Z, Gould A, Coupar B. 1995. Antigen specificity of the ovine cytotoxic T lymphocyte response to bluetongue virus. Vet Immunol Immunopathol 47:311–322. 10.1016/0165-2427(94)05410-t. 8571549

[17] Jeggo MH, Wardley RC, Brownlie J. 1985. Importance of ovine cytotoxic T cells in protection against bluetongue virus infection. Prog Clin Biol Res 178:477–487. 2989889

[18] Boyce M, Celma CCP, Roy P. 2012. Bluetongue virus non-structural protein 1 is a positive regulator of viral protein synthesis. Virol J 9:178. 10.1186/1743-422X-9-178. 22931514 PMC3479040

[19] Owens RJ, Limn C, Roy P. 2004. Role of an arbovirus nonstructural protein in cellular pathogenesis and virus release. J Virol 78:6649–6656. 10.1128/JVI.78.12.6649-6656.2004. 15163755 PMC416502

[20] Maan S, Maan NS, Ross-Smith N, Batten CA, Shaw AE, Anthony SJ, Samuel AR, Darpel KE, Veronesi E, Oura CAL, Singh KP, Nomikou K, Potgieter AC, Attoui H, van Rooij E, van Rijn P, De Clercq K, Vandenbussche F, Zientara S, Bréard E, Sailleau C, Beer M, Hoffman B, Mellor PS, Mertens PPC. 2008. Sequence analysis of bluetongue virus serotype 8 from the Netherlands 2006 and comparison to other European strains. Virology 377:308–318. 10.1016/j.virol.2008.04.028. 18570969

[21] Roy P. 1990. Use of baculovirus expression vectors: development of diagnostic reagents, vaccines and morphological counterparts of bluetongue virus. FEMS Microbiol Immunol 2:223–234. 10.1111/j.1574-6968.1990.tb03523.x. 2178355

[22] Marín-López A, Calvo-Pinilla E, Barriales D, Lorenzo G, Brun A, Anguita J, Ortego J. 2018. CD8 T cell responses to an immunodominant epitope within the nonstructural protein NS1 provide wide immunoprotection against bluetongue virus in IFNAR ^−/−^ mice. J Virol 92:e00938-18. 10.1128/JVI.00938-18. 29875250 PMC6069212

[23] Rojas JM, Peña L, Martín V, Sevilla N. 2014. Ovine and murine T cell epitopes from the non-structural protein 1 (NS1) of bluetongue virus serotype 8 (BTV-8) are shared among viral serotypes. Vet Res 45:30. 10.1186/1297-9716-45-30. 24621015 PMC3995764

[24] Anderson J, Bréard E, Lövgren Bengtsson K, Grönvik K-O, Zientara S, Valarcher J-F, Hägglund S. 2014. Purification, stability, and immunogenicity analyses of five bluetongue virus proteins for use in development of a subunit vaccine that allows differentiation of infected from vaccinated animals. Clin Vaccine Immunol 21:443–452. 10.1128/CVI.00776-13. 24451327 PMC3957662

[25] Jones LD, Chuma T, Hails R, Williams T, Roy P. 1996. The non-structural proteins of bluetongue virus are a dominant source of cytotoxic T cell peptide determinants. J Gen Virol 77:997–1003. 10.1099/0022-1317-77-5-997. 8609498

[26] Jones LD, Williams T, Bishop D, Roy P. 1997. Baculovirus-expressed nonstructural protein NS2 of bluetongue virus induces a cytotoxic T-cell response in mice which affords partial protection. Clin Diagn Lab Immunol 4:297–301. 10.1128/cdli.4.3.297-301.1997. 9144367 PMC170522

[27] Marín-López A, Calvo-Pinilla E, Barriales D, Lorenzo G, Benavente J, Brun A, Martínez-Costas JM, Ortego J. 2017. Microspheres-prime/rMVA-boost vaccination enhances humoral and cellular immune response in IFNAR(−/−) mice conferring protection against serotypes 1 and 4 of bluetongue virus. Antiviral Res 142:55–62. 10.1016/j.antiviral.2017.03.010. 28322923

[28] Marín-López A, Otero-Romero I, de la Poza F, Menaya-Vargas R, Calvo-Pinilla E, Benavente J, Martínez-Costas JM, Ortego J. 2014. VP2, VP7, and NS1 proteins of bluetongue virus targeted in avian reovirus muNS-Mi microspheres elicit a protective immune response in IFNAR(-/-) mice. Antiviral Res 110:42–51. 10.1016/j.antiviral.2014.07.008. 25057758

[29] Sheehy SH, Duncan CJ, Elias SC, Collins KA, Ewer KJ, Spencer AJ, Williams AR, Halstead FD, Moretz SE, Miura K, Epp C, Dicks MD, Poulton ID, Lawrie AM, Berrie E, Moyle S, Long CA, Colloca S, Cortese R, Gilbert SC, Nicosia A, Hill AV, Draper SJ. 2011. Phase Ia clinical evaluation of the plasmodium falciparum blood-stage antigen MSP1 in ChAd63 and MVA vaccine vectors. Mol Ther 19:2269–2276. 10.1038/mt.2011.176. 21862998 PMC3242658

[30] Wilkie M, Satti I, Minhinnick A, Harris S, Riste M, Ramon RL, Sheehan S, Thomas Z-RM, Wright D, Stockdale L, Hamidi A, O'Shea MK, Dwivedi K, Behrens HM, Davenne T, Morton J, Vermaak S, Lawrie A, Moss P, McShane H. 2020. A phase I trial evaluating the safety and immunogenicity of a candidate tuberculosis vaccination regimen, ChAdOx1 85A prime – MVA85A boost in healthy UK adults. Vaccine 38:779–789. 10.1016/j.vaccine.2019.10.102. 31735500 PMC6985898

[31] Ewer K, Rampling T, Venkatraman N, Bowyer G, Wright D, Lambe T, Imoukhuede EB, Payne R, Fehling SK, Strecker T, Biedenkopf N, Krähling V, Tully CM, Edwards NJ, Bentley EM, Samuel D, Labbé G, Jin J, Gibani M, Minhinnick A, Wilkie M, Poulton I, Lella N, Roberts R, Hartnell F, Bliss C, Sierra-Davidson K, Powlson J, Berrie E, Tedder R, Roman F, De Ryck I, Nicosia A, Sullivan NJ, Stanley DA, Mbaya OT, Ledgerwood JE, Schwartz RM, Siani L, Colloca S, Folgori A, Di Marco S, Cortese R, Wright E, Becker S, Graham BS, Koup RA, Levine MM, Volkmann A, Chaplin P, et al. 2016. A monovalent chimpanzee adenovirus Ebola vaccine boosted with MVA. N Engl J Med 374:1635–1646. 10.1056/NEJMoa1411627. 25629663 PMC5798586

[32] Graham SP, McLean RK, Spencer AJ, Belij-Rammerstorfer S, Wright D, Ulaszewska M, Edwards JC, Hayes JWP, Martini V, Thakur N, Conceicao C, Dietrich I, Shelton H, Waters R, Ludi A, Wilsden G, Browning C, Bialy D, Bhat S, Stevenson-Leggett P, Hollinghurst P, Gilbride C, Pulido D, Moffat K, Sharpe H, Allen E, Mioulet V, Chiu C, Newman J, Asfor AS, Burman A, Crossley S, Huo J, Owens RJ, Carroll M, Hammond JA, Tchilian E, Bailey D, Charleston B, Gilbert SC, Tuthill TJ, Lambe T. 2020. Evaluation of the immunogenicity of prime-boost vaccination with the replication-deficient viral vectored COVID-19 vaccine candidate ChAdOx1 nCoV-19. 1. NPJ Vaccines 5:1–6. 10.1038/s41541-020-00221-3. 32793398 PMC7385486

[33] Folegatti PM, Ewer KJ, Aley PK, Angus B, Becker S, Belij-Rammerstorfer S, Bellamy D, Bibi S, Bittaye M, Clutterbuck EA, Dold C, Faust SN, Finn A, Flaxman AL, Hallis B, Heath P, Jenkin D, Lazarus R, Makinson R, Minassian AM, Pollock KM, Ramasamy M, Robinson H, Snape M, Tarrant R, Voysey M, Green C, Douglas AD, Hill AVS, Lambe T, Gilbert SC, Pollard AJ, Aboagye J, Adams K, Ali A, Allen E, Allison JL, Anslow R, Arbe-Barnes EH, Babbage G, Baillie K, Baker M, Baker N, Baker P, Baleanu I, Ballaminut J, Barnes E, Barrett J, Bates L, Batten A, et al. 2020. Safety and immunogenicity of the ChAdOx1 nCoV-19 vaccine against SARS-CoV-2: a preliminary report of a phase 1/2, single-blind, randomised controlled trial. Lancet 396:467–478. 10.1016/S0140-6736(20)31604-4. 32702298 PMC7445431

[34] Alharbi NK, Qasim I, Almasoud A, Aljami HA, Alenazi MW, Alhafufi A, Aldibasi OS, Hashem AM, Kasem S, Albrahim R, Aldubaib M, Almansour A, Temperton NJ, Kupke A, Becker S, Abu-Obaidah A, Alkarar A, Yoon I-K, Azhar E, Lambe T, Bayoumi F, Aldowerij A, Ibrahim OH, Gilbert SC, Balkhy HH. 2019. Humoral immunogenicity and efficacy of a single dose of ChAdOx1 MERS vaccine candidate in dromedary camels. Sci Rep 9 10.1038/s41598-019-52730-4. PMC6841732 31705137

[35] Warimwe GM, Gesharisha J, Carr BV, Otieno S, Otingah K, Wright D, Charleston B, Okoth E, Elena L-G, Lorenzo G, Ayman E-B, Alharbi NK, Al-Dubaib MA, Brun A, Gilbert SC, Nene V, Hill AVS. 2016. Chimpanzee Adenovirus vaccine provides multispecies protection against Rift Valley Fever. Sci Rep 6:20617–20617. 10.1038/srep20617. 26847478 PMC4742904

[36] Antrobus RD, Coughlan L, Berthoud TK, Dicks MD, Hill AV, Lambe T, Gilbert SC. 2014. Clinical assessment of a novel recombinant simian Adenovirus ChAdOx1 as a vectored vaccine expressing conserved Influenza A antigens. Mol Ther 22:668–674. 10.1038/mt.2013.284. 24374965 PMC3944330

[37] van Doremalen N, Lambe T, Spencer A, Belij-Rammerstorfer S, Purushotham JN, Port JR, Avanzato VA, Bushmaker T, Flaxman A, Ulaszewska M, Feldmann F, Allen ER, Sharpe H, Schulz J, Holbrook M, Okumura A, Meade-White K, Pérez-Pérez L, Edwards NJ, Wright D, Bissett C, Gilbride C, Williamson BN, Rosenke R, Long D, Ishwarbhai A, Kailath R, Rose L, Morris S, Powers C, Lovaglio J, Hanley PW, Scott D, Saturday G, de Wit E, Gilbert SC, Munster VJ. 2020. ChAdOx1 nCoV-19 vaccine prevents SARS-CoV-2 pneumonia in rhesus macaques. Nature 586:578–582. 10.1038/s41586-020-2608-y. 32731258 PMC8436420

[38] Dicks MDJ, Spencer AJ, Edwards NJ, Wadell G, Bojang K, Gilbert SC, Hill AVS, Cottingham MG. 2012. A novel chimpanzee adenovirus vector with low human seroprevalence: improved systems for vector derivation and comparative immunogenicity. PLoS One 7:e40385. 10.1371/journal.pone.0040385. 22808149 PMC3396660

[39] Utrilla-Trigo S, Jiménez-Cabello L, Alonso-Ravelo R, Calvo-Pinilla E, Marín-López A, Moreno S, Lorenzo G, Benavides J, Gilbert S, Nogales A, Ortego J. 2020. Heterologous combination of ChAdOx1 and MVA vectors expressing protein NS1 as vaccination strategy to induce durable and cross-protective CD8+ T Cell immunity to bluetongue virus. Vaccines 8:346. 10.3390/vaccines8030346. PMC7564706 32610561

[40] Calvo-Pinilla E, Marín-López A, Moreno S, Lorenzo G, Utrilla-Trigo S, Jiménez-Cabello L, Benavides J, Nogales A, Blasco R, Brun A, Ortego J. 2020. A protective bivalent vaccine against Rift Valley fever and bluetongue. NPJ Vaccines 5:1–12. 10.1038/s41541-020-00218-y. 32793399 PMC7393076

[41] Howerth EW, Greene CE, Prestwood AK. 1988. Experimentally induced bluetongue virus infection in white-tailed deer: coagulation, clinical pathologic, and gross pathologic changes. Am J Vet Res 49:1906–1913. 2854709

[42] Anderson J, Hägglund S, Bréard E, Comtet L, Lövgren Bengtsson K, Pringle J, Zientara S, Valarcher JF. 2013. Evaluation of the immunogenicity of an experimental subunit vaccine that allows differentiation between infected and vaccinated animals against bluetongue virus serotype 8 in cattle. Clin Vaccine Immunol 20:1115–1122. 10.1128/CVI.00229-13. 23720365 PMC3754508

[43] Anderson J, Hägglund S, Bréard E, Riou M, Zohari S, Comtet L, Olofson A-S, Gélineau R, Martin G, Elvander M, Blomqvist G, Zientara S, Valarcher JF. 2014. Strong protection induced by an experimental DIVA subunit vaccine against bluetongue virus serotype 8 in cattle. Vaccine 32:6614–6621. 10.1016/j.vaccine.2014.09.066. 25312275

[44] Parga-Vidal L, van Gisbergen KPJM. 2020. Area under immunosurveillance: dedicated roles of memory CD8 T-cell subsets. Cold Spring Harb Perspect Biol 12:a037796. 10.1101/cshperspect.a037796. 32839203 PMC7605223

[45] Bachmann MF, Wolint P, Schwarz K, Jäger P, Oxenius A. 2005. Functional properties and lineage relationship of CD8+ T cell subsets identified by expression of IL-7 receptor alpha and CD62L. J Immunol 175:4686–4696. 10.4049/jimmunol.175.7.4686. 16177116

[46] Wherry EJ, Teichgräber V, Becker TC, Masopust D, Kaech SM, Antia R, von Andrian UH, Ahmed R. 2003. Lineage relationship and protective immunity of memory CD8 T cell subsets. Nat Immunol 4:225–234. 10.1038/ni889. 12563257

[47] Stanley DA, Honko AN, Asiedu C, Trefry JC, Lau-Kilby AW, Johnson JC, Hensley L, Ammendola V, Abbate A, Grazioli F, Foulds KE, Cheng C, Wang L, Donaldson MM, Colloca S, Folgori A, Roederer M, Nabel GJ, Mascola J, Nicosia A, Cortese R, Koup RA, Sullivan NJ. 2014. Chimpanzee adenovirus vaccine generates acute and durable protective immunity against ebolavirus challenge. Nat Med 20:1126–1129. 10.1038/nm.3702. 25194571

[48] Fischer RJ, Purushotham JN, van Doremalen N, Sebastian S, Meade-White K, Cordova K, Letko M, Jeremiah Matson M, Feldmann F, Haddock E, LaCasse R, Saturday G, Lambe T, Gilbert SC, Munster VJ. 2021. ChAdOx1-vectored Lassa fever vaccine elicits a robust cellular and humoral immune response and protects guinea pigs against lethal Lassa virus challenge. NPJ Vaccines 6:32. 10.1038/s41541-021-00291-x. 33654106 PMC7925663

[49] Munster VJ, Wells D, Lambe T, Wright D, Fischer RJ, Bushmaker T, Saturday G, van Doremalen N, Gilbert SC, de Wit E, Warimwe GM. 2017. Protective efficacy of a novel simian adenovirus vaccine against lethal MERS-CoV challenge in a transgenic human DPP4 mouse model. NPJ Vaccines 2:1–4. 10.1038/s41541-017-0029-1. 29263883 PMC5643297

[50] Warimwe GM, Lorenzo G, Lopez-Gil E, Reyes-Sandoval A, Cottingham MG, Spencer AJ, Collins KA, Dicks MDJ, Milicic A, Lall A, Furze J, Turner AV, Hill AVS, Brun A, Gilbert SC. 2013. Immunogenicity and efficacy of a chimpanzee adenovirus-vectored Rift Valley fever vaccine in mice. Virol J 10:349. 10.1186/1743-422X-10-349. 24304565 PMC4235025

[51] López-Camacho C, Abbink P, Larocca RA, Dejnirattisai W, Boyd M, Badamchi-Zadeh A, Wallace ZR, Doig J, Velazquez RS, Neto RDL, Coelho DF, Kim YC, Donald CL, Owsianka A, De Lorenzo G, Kohl A, Gilbert SC, Dorrell L, Mongkolsapaya J, Patel AH, Screaton GR, Barouch DH, Hill AVS, Reyes-Sandoval A. 2018. Rational Zika vaccine design via the modulation of antigen membrane anchors in chimpanzee adenoviral vectors. Nat Commun 9 10.1038/s41467-018-04859-5. PMC6015009 29934593

[52] Sánchez-Cordón PJ, Pleguezuelos FJ, Pérez de Diego AC, Gómez-Villamandos JC, Sánchez-Vizcaíno JM, Cerón JJ, Tecles F, Garfia B, Pedrera M. 2013. Comparative study of clinical courses, gross lesions, acute phase response and coagulation disorders in sheep inoculated with bluetongue virus serotype 1 and 8. Vet Microbiol 166:184–194. 10.1016/j.vetmic.2013.05.032. 23849094

[53] Worwa G, Hilbe M, Chaignat V, Hofmann MA, Griot C, Ehrensperger F, Doherr MG, Thür B. 2010. Virological and pathological findings in bluetongue virus serotype 8 infected sheep. Vet Microbiol 144:264–273. 10.1016/j.vetmic.2010.01.011. 20153937

[54] Flanagan M, Wilson AJ, Trueman KF, Shepherd MA. 1982. Bluetongue virus serotype 20 infection in pregnant Merino sheep. Aust Vet J 59:18–20. 10.1111/j.1751-0813.1982.tb02704.x. 6293437

[55] Swadling L, Capone S, Antrobus RD, Brown A, Richardson R, Newell EW, Halliday J, Kelly C, Bowen D, Fergusson J, Kurioka A, Ammendola V, Del Sorbo M, Grazioli F, Esposito ML, Siani L, Traboni C, Hill A, Colloca S, Davis M, Nicosia A, Cortese R, Folgori A, Klenerman P, Barnes E. 2014. A human vaccine strategy based on chimpanzee adenoviral and MVA vectors that primes, boosts, and sustains functional HCV-specific T cell memory. Sci Transl Med 6:261ra153. 10.1126/scitranslmed.3009185. PMC4669853 25378645

[56] Green C, Scarselli E, Sande C, Thompson A, de Lara C, Taylor K, Haworth K, Del Sorbo M, Angus B, Siani L, Di Marco S, Traboni C, Folgori A, Colloca S, Capone S, Vitelli A, Cortese R, Klenerman P, Nicosia A, Pollard A. 2015. Chimpanzee adenovirus and MVA-vectored respiratory syncytial virus vaccine is safe and expands humoral and cellular immunity in adults. Sci Transl Med 7:300ra126. 10.1126/scitranslmed.aac5745 PMC4669850 26268313

[57] Capone S, Brown A, Hartnell F, Sorbo MD, Traboni C, Vassilev V, Colloca S, Nicosia A, Cortese R, Folgori A, Klenerman P, Barnes E, Swadling L. 2020. Optimising T cell (re)boosting strategies for adenoviral and modified vaccinia Ankara vaccine regimens in humans. NPJ Vaccines 5:1–14. 10.1038/s41541-020-00240-0. 33083029 PMC7550607

[58] Barros-Martins J, Hammerschmidt SI, Cossmann A, Odak I, Stankov MV, Morillas Ramos G, Dopfer-Jablonka A, Heidemann A, Ritter C, Friedrichsen M, Schultze-Florey C, Ravens I, Willenzon S, Bubke A, Ristenpart J, Janssen A, Ssebyatika G, Bernhardt G, Münch J, Hoffmann M, Pöhlmann S, Krey T, Bošnjak B, Förster R, Behrens GMN. 2021. Immune responses against SARS-CoV-2 variants after heterologous and homologous ChAdOx1 nCoV-19/BNT162b2 vaccination. Nat Med 27:1525–1529. 10.1038/s41591-021-01449-9. 34262158 PMC8440184

[59] Spencer AJ, McKay PF, Belij-Rammerstorfer S, Ulaszewska M, Bissett CD, Hu K, Samnuan K, Blakney AK, Wright D, Sharpe HR, Gilbride C, Truby A, Allen ER, Gilbert SC, Shattock RJ, Lambe T. 2021. Heterologous vaccination regimens with self-amplifying RNA and adenoviral COVID vaccines induce robust immune responses in mice. Nat Commun 12:2893. 10.1038/s41467-021-23173-1. 34001897 PMC8129084

[60] Schmidt T, Klemis V, Schub D, Mihm J, Hielscher F, Marx S, Abu-Omar A, Ziegler L, Guckelmus C, Urschel R, Schneitler S, Becker SL, Gärtner BC, Sester U, Sester M. 2021. Immunogenicity and reactogenicity of heterologous ChAdOx1 nCoV-19/mRNA vaccination. Nat Med 27:1530–1535. 10.1038/s41591-021-01464-w. 34312554 PMC8440177

[61] Chinnakannan SK, Cargill TN, Donnison TA, Ansari MA, Sebastian S, Lee LN, Hutchings C, Klenerman P, Maini MK, Evans T, Barnes E. 2020. The design and development of a multi-HBV antigen encoded in chimpanzee adenoviral and modified vaccinia Ankara viral vectors; a novel therapeutic vaccine strategy against HBV. Vaccines 8:184. 10.3390/vaccines8020184. PMC7348829 32295168

[62] Tully CM, Chinnakannan S, Mullarkey CE, Ulaszewska M, Ferrara F, Temperton N, Gilbert SC, Lambe T. 2017. Novel bivalent viral-vectored vaccines induce potent humoral and cellular immune responses conferring protection against stringent Influenza A virus challenge. J Immunol Epub ahead of print. . 10.4049/jimmunol.1600939. 28724579

[63] Bliss CM, Bowyer G, Anagnostou NA, Havelock T, Snudden CM, Davies H, de Cassan SC, Grobbelaar A, Lawrie AM, Venkatraman N, Poulton ID, Roberts R, Mange PB, Choudhary P, Faust SN, Colloca S, Gilbert SC, Nicosia A, Hill AVS, Ewer KJ. 2018. Assessment of novel vaccination regimens using viral vectored liver stage malaria vaccines encoding ME-TRAP. Sci Rep 8:3390. 10.1038/s41598-018-21630-4. 29467399 PMC5821890

[64] Dungu B, Gerdes T, Smit T. 2004. The use of vaccination in the control of bluetongue in southern Africa. Vet Ital 40:616–622. 20422597

[65] van Rijn PA, Maris-Veldhuis MA, Spedicato M, Savini G, van Gennip RGP. 2021. Pentavalent disabled infectious single animal (DISA)/DIVA vaccine provides protection in sheep and cattle against different serotypes of bluetongue virus. Vaccines 9:1150. 10.3390/vaccines9101150. 34696258 PMC8537505

[66] Kardani K, Bolhassani A, Shahbazi S. 2016. Prime-boost vaccine strategy against viral infections: mechanisms and benefits. Vaccine 34:413–423. 10.1016/j.vaccine.2015.11.062. 26691569

[67] Yap KL, Ada GL, McKenzie IF. 1978. Transfer of specific cytotoxic T lymphocytes protects mice inoculated with influenza virus. Nature 273:238–239. 10.1038/273238a0. 306072

[68] Webster RG, Askonas BA. 1980. Cross-protection and cross-reactive cytotoxic T cells induced by influenza virus vaccines in mice. Eur J Immunol 10:396–401. 10.1002/eji.1830100515. 6967815

[69] Bachmann MF, Speiser DE, Ohashi PS. 1997. Functional management of an antiviral cytotoxic T-cell response. J Virol 71:5764–5768. 10.1128/JVI.71.8.5764-5768.1997. 9223463 PMC191829

[70] Flynn KJ, Belz GT, Altman JD, Ahmed R, Woodland DL, Doherty PC. 1998. Virus-specific CD8+ T cells in primary and secondary influenza pneumonia. Immunity 8:683–691. 10.1016/s1074-7613(00)80573-7. 9655482

[71] McMichael AJ. 2018. Is a human CD8 T-Cell Vaccine possible, and if so, what would it take? Cold Spring Harb Perspect Biol 10:a029124. 10.1101/cshperspect.a029124. 29254977 PMC6005744

[72] Veronesi E, Darpel K, Gubbins S, Batten C, Nomikou K, Mertens P, Carpenter S. 2020. Diversity of transmission outcomes following co-infection of sheep with strains of bluetongue virus serotype 1 and 8. Microorganisms 8:851. 10.3390/microorganisms8060851. PMC7356686 32516979

[73] Melzi E, Caporale M, Rocchi M, Martín V, Gamino V, di Provvido A, Marruchella G, Entrican G, Sevilla N, Palmarini M. 2016. Follicular dendritic cell disruption as a novel mechanism of virus-induced immunosuppression. Proc Natl Acad Sci USA 113:E6238–E6247. 10.1073/pnas.1610012113. 27671646 PMC5068271

[74] van Gennip RGP, Drolet BS, Rozo Lopez P, Roost AJC, Boonstra J, van Rijn PA. 2019. Vector competence is strongly affected by a small deletion or point mutations in bluetongue virus. Parasit Vectors 12:470. 10.1186/s13071-019-3722-2. 31604476 PMC6790033

[75] Feenstra F, Drolet BS, Boonstra J, van Rijn PA. 2015. Non-structural protein NS3/NS3a is required for propagation of bluetongue virus in Culicoides sonorensis. Parasit Vectors 8:476. 10.1186/s13071-015-1063-3. 26383094 PMC4573936

[76] Federici V, Goffredo M, Mancini G, Quaglia M, Santilli A, Di Nicola F, De Ascentis M, Cabras P, Volpicelli C, De Liberato C, Satta G, Federico G, Leone A, Pisciella M, Portanti O, Pizzurro F, Teodori L, Savini G. 2019. Vector competence of Italian populations of Culicoides for some bluetongue virus strains responsible for recent Northern African and European outbreaks. Viruses 11:941. 10.3390/v11100941. PMC6832517 31614799

[77] Nomikou K, Hughes J, Wash R, Kellam P, Breard E, Zientara S, Palmarini M, Biek R, Mertens P. 2015. Widespread reassortment shapes the evolution and epidemiology of bluetongue virus following European invasion. PLoS Pathog 11:e1005056. 10.1371/journal.ppat.1005056. 26252219 PMC4529188

[78] Marín-López A, Bermúdez R, Calvo-Pinilla E, Moreno S, Brun A, Ortego J. 2016. Pathological characterization Of IFNAR(-/-) mice infected with bluetongue virus serotype 4. Int J Biol Sci 12:1448–1460. 10.7150/ijbs.14967. 27994510 PMC5166487

[79] Calvo-Pinilla E, Rodríguez-Calvo T, Sevilla N, Ortego J. 2009. Heterologous prime boost vaccination with DNA and recombinant modified vaccinia virus Ankara protects IFNAR(−/−) mice against lethal bluetongue infection. Vaccine 28:437–445. 10.1016/j.vaccine.2009.10.027. 19857449

[80] Marín-López A, Ortego J. 2016. Generation of recombinant modified vaccinia virus Ankara encoding VP2, NS1, and VP7 proteins of bluetongue virus, p 137–150. In Brun A (ed), Vaccine technologies for veterinary viral diseases. Springer New York, New York, NY. 10.1007/978-1-4939-3008-1_9 26458834

[81] Calvo-Pinilla E, Navasa N, Anguita J, Ortego J. 2012. Multiserotype protection elicited by a combinatorial prime-boost vaccination strategy against bluetongue virus. PLoS One 7:e34735. 10.1371/journal.pone.0034735. 22514660 PMC3326038

[82] Sánchez-Puig JM, Blasco R. 2005. Isolation of vaccinia MVA recombinants using the viral F13L gene as the selective marker. Biotechniques 39:665–674. 10.2144/000112012. 16312215

[83] Toussaint JF, Sailleau C, Breard E, Zientara S, De Clercq K. 2007. Bluetongue virus detection by two real-time RT-qPCRs targeting two different genomic segments. J Virol Methods 140:115–123. 10.1016/j.jviromet.2006.11.007. 17196266

[84] Perrin A, Albina E, Bréard E, Sailleau C, Promé S, Grillet C, Kwiatek O, Russo P, Thiéry R, Zientara S, Cêtre-Sossah C. 2007. Recombinant capripox viruses expressing proteins of bluetongue virus: Evaluation of immune responses and protection in small ruminants. Vaccine 25:6774–6783. 10.1016/j.vaccine.2007.06.052. 17669563

