# Supplementary material for: The Combined Expression of the Nonstructural Protein NS1 and the N-Terminal Half of NS2 (NS21-180) by ChAdOx1 and MVA Confers Protection against Clinical Disease in Sheep upon Bluetongue Virus Challenge
Source: J Virol. 2022 Feb 9;96(3):e01614-21. doi: 10.1128/JVI.01614-21 (PMC8826911; doi:10.1128/JVI.01614-21)
Supplement: Supplemental file 1 — Fig. S1. Download JVI.01614-21-s0001.pdf, PDF file, 0.5 MB [file jvi.01614-21-s0001.pdf]

Supplementary Figure 1

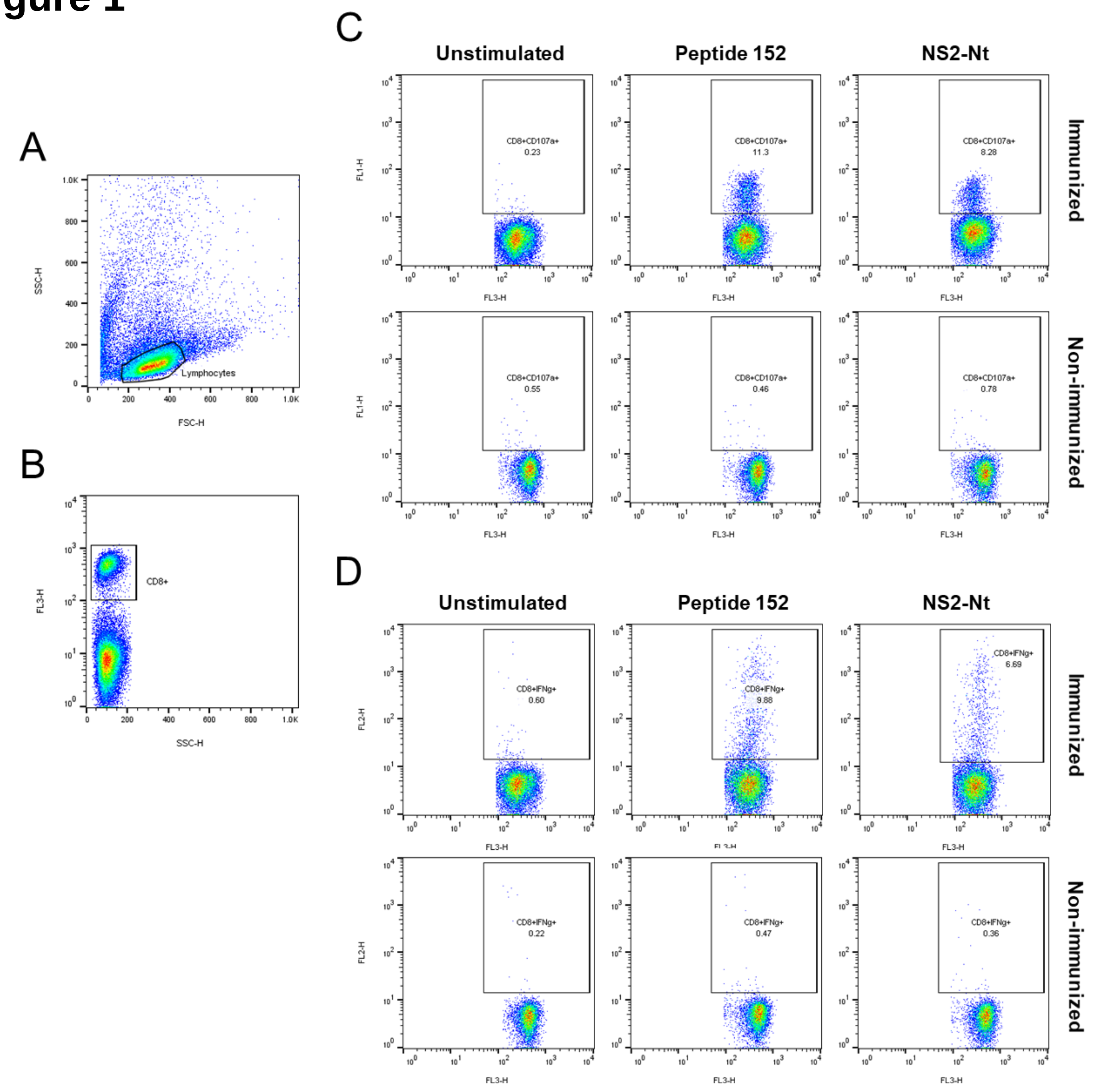

Supplementary Figure 1. Gating strategy used to identify CD8+ T cell populations expressing CD107a or secreting IFN-γ in response to BTV antigens. Splenocytes from MVA/ChAdOx1-NS1-NS2-Nt immunized and mock-immunized mice were stimulated with peptide 152(NS1) or NS2-Nt protein. After polychromatic staining of cells with different fluorochrome-conjugated antibodies, flow cytometric analyses were carried out. Lymphocytes were first gated using FSC and SSC (A) and then gated on the CD8+ cell population (B) using CD8-PerCp-Vio700 antibody. Panels show an example of flow cytometric analyses for CD107a degranulation marker (C) and IFN-γ (D) production in CD8+ T cell subsets using CD107a-FITC and IFN-γ-PE antibodies in a non-vaccinated mouse or a ChAdOx1/MVA-NS1-NS2-Nt vaccinated mouse. Results of the analyses of each group of mice are presented in Figure 5 of the manuscript.
